# High Entropy Oxides: Mapping the Landscape from Fundamentals to Future Vistas: Focus Review

**DOI:** 10.1021/acsenergylett.4c01129

**Published:** 2024-07-05

**Authors:** Suvodeep Sen, Manoj Palabathuni, Kevin M. Ryan, Shalini Singh

**Affiliations:** Department of Chemical Sciences and Bernal Institute, University of Limerick, V94 T9PX Limerick, Ireland

## Abstract

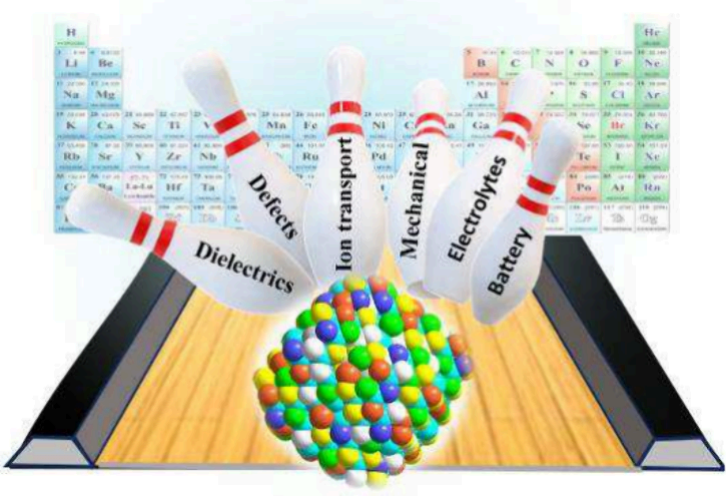

High-entropy materials (HEMs) are typically crystalline, phase-pure and configurationally disordered materials that contain at least five elements evenly blended into a solid-solution framework. The discovery of high-entropy alloys (HEAs) and high-entropy oxides (HEOs) disrupted traditional notions in materials science, providing avenues for the exploration of new materials, property optimization, and the pursuit of advanced applications. While there has been significant research on HEAs, the creative breakthroughs in HEOs are still being revealed. This focus review aims at developing a structured framework for expressing the concept of HEM, with special emphasis on the crystal structure and functional properties of HEOs. Insights into the recent synthetic advances, that foster prospective outcomes and their current applications in electrocatalysis, and battery, are comprehensively discussed. Further, it sheds light on the existing constraints in HEOs, highlights the adoption of theoretical and experimental tools to tackle challenges, while delineates potential directions for exploration in energy application.

Alloying boasts a rich historical legacy owing to its ability to impact desirable properties for materials.^1,2^ It usually entails the incorporation of smaller amounts of secondary elements into a principal or primary element.^3,4^ Such methodology enables the creation of finite varieties of potential alloys, and numerous studies in the past have thoroughly investigated these alloy possibilities. This prevailing influence arises from the typically restricted miscibility of elements.^4^ High-entropy alloys (HEAs) are an unorthodox class of emerging materials that have thrived during the current decade.^5,6^ They mostly contain five or more principal elements with nearly equimolar compositions, that utilize a high configurational entropy to stabilize multiple elements within a single crystal lattice or sublattice.^7,8^ The multielemental composition and homogeneously mixed solid-solution (SS) state in high-entropy materials (HEMs) not only offer an extensive array of material combinations for discovery but also present a distinctive microstructure for optimizing properties.^9−11^ The first reports on multicomponent and crystalline HEAs were published in 2004, by Yeh and Cantor.^12,13^ They proposed that higher-order alloys can be achieved by increasing the configurational entropy of mixing beyond the enthalpy associated with compound formation. However, these seminal reports did not furnish a detailed structural understanding, nor did they unveiled any general synthetic pathway for various compositions. But, in 2016, Mirkin and co-workers achieved a remarkable breakthrough in synthesizing diverse compositions of multielemental, but phase-segregated, nanoparticles (NPs) using confined nanoreactors.^3^ Since then, the research on the development of HEMs has risen to prominence, demonstrating improved mechanical properties and holding promise for a broad spectrum of possible applications, including but not limited to catalysis,^14−17^ battery,^18−20^ refractory,^21^ thermoelectrics,^22,23^ hydrogen storage,^24,25^ and cryogenic technologies.^26^ The in-depth examination of HEMs has become achievable due to the swift progress in synthetic methodologies,^27,28^ advanced characterization techniques,^29^ high-throughput experimentation, and data-driven discoveries.^30,31^ However, the multidimensional compositional space that can be addressed with these materials is boundless, with very small regions having been explored thus far.

Advancement of HEAs instilled the researchers with the quest for discovery of other classes of HEMs.^32−37^ And gradually, they also adopted the terminology “high-entropy (HE)” to outline the different material systems including HE oxides and sulfides, among others. The emergence of high-entropy oxides (HEOs) challenged the conventional materials science by seeking to understand the unique properties that unfold from significant configurational disorder, presenting a paradigm shift in our understanding of materials.^38^ The limitless opportunities for energy-related processes, notably in catalytic energy conversion and storage, have sparked widespread interest in harnessing the potential of HEOs.^5,39^ The multielemental surface of HEOs exhibits a variety of distinct adsorption sites in proximity, making it desirable for multistep reactions involving multiple electron transfers. While the electronic landscape can be tuned by adjusting the material stoichiometry, the SS mixing offers potential structural stability that is critical to functioning under harsh environmental conditions. This tunability enables customization for specific reactions and types of products. Besides, undesirable elements, whether toxic, deficient, or expensive, can be substituted with a blend of other elements possessing similar properties.

In this Focus Review, we delve into the underlying motivations, encompassing fundamentals, synthesis innovations, and applied aspects of HEOs, that are propelling this class of materials into the realm of being considered as the most growing area of research. A summary of the Focus Review, encompassing synthesis, computation, and throughput characterization of their properties, applications, and future development, is depicted through the schematic in Figure 1. We initially establish the framework with a rational understanding of HEMs in general and HEOs in particular, from both the thermodynamic and kinetic standpoint. The recent progress in the synthetic innovations of HEOs and their applications breakthroughs in catalysis and battery applications are detailed in this Focus Review, while the critical needs for the development of these enticing materials for diverse applications are comprehensively discussed. In a nutshell, this Focus Review adopts a multidisciplinary approach to offer cutting-edge insights derived from the latest research, exploring the mechanisms through which HEOs, or the general HE NPs, take shape. Additionally, it explores the consequential impact of these formation pathways on crucial features of NPs and their functionality. Finally, it concludes with discussing the advanced fundamental understanding and experimental and computational breakthroughs that can mitigate the pressing needs in these materials, while anticipating their impact in prospective applications.

**Figure 1:**
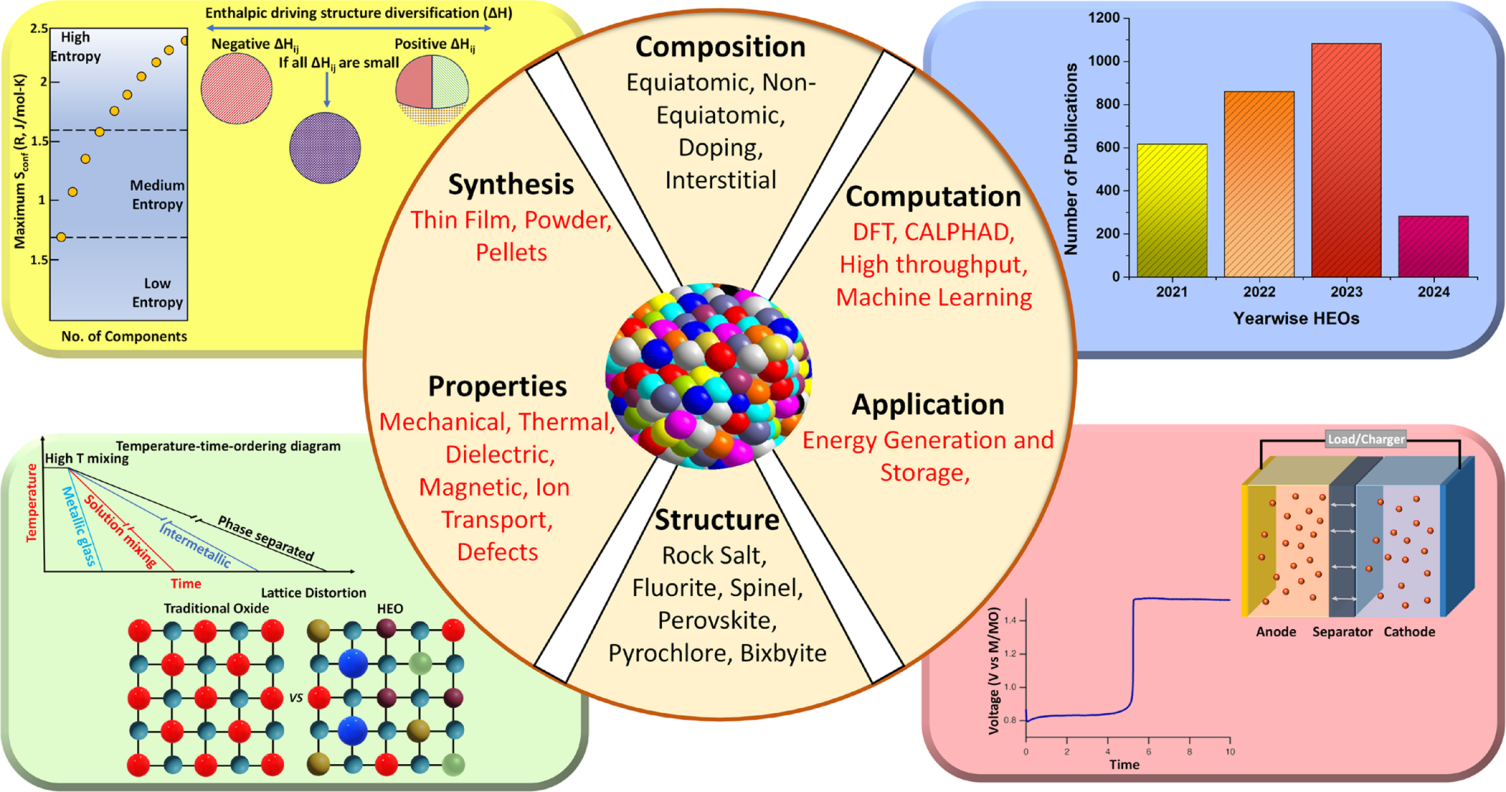
Schematic illustrating of the synthesis of HEOs, their computational simulation aided material design, properties, and applications. The values mentioned for each bar of the histogram (top left panel) were acquired with keywords in the Web of Knowledge search portal on 31st March 2024.

## ENTROPY’S SPECTRUM: HIGH-ENTROPY CONCEPTS EXPLORED

The existence of multiple definitions for HEAs contributes to confusion and controversy regarding which alloys qualify as such. Owing to their recent emergence, a universally accepted definition of HEOs has not been established yet. Thus, HEOs can be characterized as single-phase oxide systems with five or more cations, where the configurational entropy (*S*_*config*_) exceeds 1.5*R* (here *R* = 8.314 J K^–1^ mol^–1^ is the universal gas constant). However, in the context of oxide materials, the three essential requisites, namely, crystallinity, phase purity, and configurational disorder, discussed above are satisfied by numerous traditional SS states.^40^ Therefore, additional yardsticks are needed to distinguish HEOs from conventional oxides.^41−43^ In this segment, we will discuss the concept of HE through the interplay of thermodynamic and kinetic parameters. The discussions will briefly include the basic thermodynamic concepts of entropy, enthalpy, and Gibbs energy characteristics of disordered SS phases, while also highlight how the presence of multiprincipal elements influences the magnitudes of these terms. Furthermore, we will discuss the dynamics of slow diffusion in HEOs due to kinetic factors such as reaction mechanism, temperature dependence, quenching rate, etc. and how it impacts their crystal structures and, but not limited to, mechanical properties. Thus, the objective of this segment is to understand the four core effects, viz., the high entropy effect, the lattice distortion, sluggish diffusion, and the “cocktail” effect, that better describe HEOs. In addition, we also examine the different approaches used in the literature to distinguish HEOs from conventional oxide materials.

### Power of Disorder: Thermodynamic Entropy Effect

Yeh et al. proposed the idea of HEAs by considering the rise in configurational entropy of mixing with the inclusion of equimolar principal elements in alloy systems.^44^ The presence of more and more elements leads to a plethora of plausible atomic arrangements, resulting in disorder and a high configurational entropy of mixing (Δ*S*_*mix*_). This entropy can be determined through calculation as
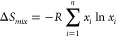
1where *x*_*i*_ denotes the concentration of component *i* and *n* is the number of elements. In oxide systems, the ideal *S*_*config*_ from the O^2–^ site is expected to be zero. But, the introduction of oxygen defects or other anions, such as F^–^ in oxyfluorides or S^2–^ in oxysulfides, can contribute additional entropy to the configuration of the anionic sublattice. When considering HEOs, this definition can be easily modified to incorporate a second summation across sublattices.
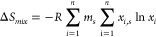
2

Here *m*_*s*_ denotes the multiplicity of sublattice *s* and *x*_*i,s*_ represent the proportion of element *i* present on sublattice s. Thus, such adjustment takes into consideration possible influences on the overall *S*_config_, stemming from various factors such as multiple cation sublattices, oxygen vacancies, or other types of disorder within the anion sublattice. Within the domain of HEMs, the historical perspective defines high entropy as Δ*S*_*mix*_ > 1.5*R*. Based on empirical observations in alloys, it has been inferred that a distinct phenomenon occurs when transitioning from four to five elements. This transition marks a critical threshold where the likelihood of achieving a homogeneous single-phase material becomes more prevalent. These benchmarks for recognizing HEAs have been directly employed to identify HEOs. But it should be noted that the Δ*S*_*mix*_ > 1.5*R* calculation is typically applicable in the context of ideal SS and might not yield accurate results due to deviations from ideality.

Another way to differentiate a HEM from a conventional SS is by the stipulation that the material must be inherently stabilized by entropy. Founded on this hypothesis, it was proposed that the thermodynamic contribution of high Δ*S*_*mix*_ in a system containing at least five elements in equimolar proportions contributes significantly to the Gibbs free energy (Δ*G*_*mix*_) at elevated temperatures. This contribution adequately offsets the enthalpy of formation (Δ*H*_*mix*_) associated with intermetallic compounds, facilitating the creation of a unified solution comprising multiple elements (Δ*G*_*mix*_ ≤ 0).
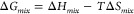
3where *T* is the absolute temperature. It should be noted that entropic contributions to free energy increase proportionally with rise in temperature. In contrast, the Δ*H*_*mix*_ remains nearly independent of temperature, establishing the true ground state under zero-temperature conditions. Thus, the formation of HEMs can be understood through the thermodynamic interplay between enthalpy and entropy. It involves a dynamic balance or competition between these two fundamental thermodynamic factors. Hence, the Δ*S*_*mix*_ of an HEM increases with increasing number of elements in the system and this phenomenon serves as a compelling catalyst, propelling the process of single-phase mixing.

### Gradual Dissemination Dynamics: Understanding the Sluggish Diffusion Phenomenon

HEOs are usually synthesized at elevated temperatures through extended reaction durations. Therefore, it is presumed that the synthesis temperature allows for the attainment of thermodynamic equilibrium, and subsequent rapid cooling from this temperature fully preserves the disordered phase at room temperature. The interaction between reaction kinetics and reversibility is crucial, especially as reversibility is often highlighted as a significant factor in entropy stabilization.

Kinetics plays an inevitable role in both phase selectivity and stability of HEOs, especially on the emergence of intermetallic phases within a given system. The correlation between different cooling rates, including both rapid and slow conditions, and their effects on both solidification and solid-state phase transformation after annealing suggests that higher cooling rates and faster kinetics lead to a decrease in the formation of different intermetallic phases. A perturbation model, involving the number of elements and temperature, was reported by Luan and co-workers^45^ to assess the stabilities of phase-pure HEMs in comparison to the potential formation of intermetallic compounds. They found that the rise in the number of elements is advantageous from an entropy perspective for the creation of a phase-pure SS. However, it concurrently adds an unfavorable enthalpic component to the overall Δ*G*_*mix*_. This underscores the notable influence of elevated temperatures on the phase stability of HEMs, as higher temperatures reduce the Gibbs free energy, thereby enhancing the stability of single-phase SS. Hence, it is conclusive that, relying on thermodynamic considerations, most single-phase HEMs that develop at elevated temperatures are not inherently stable at room temperature, and a transition to a polyphase structure is anticipated. Nevertheless, kinetic factors such as rapid cooling and the slow dynamic response of multicomponent systems during the synthesis significantly govern both the formation process and stability of phase-pure HEMs (or HEOs) at ambient temperature.

The chemical complexity of HEOs also gives rise to various conceptual challenges as the experimental reality is unquestionably less ideal. For instance, in dilute conditions, the host element serves as the solvent where solutes (additional elements) dissolve. Further, in equiatomic HEOs, distinguishing between solvent and solute is not straightforward.

### Crystal Chaos: Probing Lattice Distortion

Almost all the physical and chemical parameters governing phase stability in HEAs^7,40^ can be analogously extended to HEOs. But there are some crucial differences in HEOs that need detailed investigation. The local distortions observed in HEAs are primarily attributed to variations in atomic sizes. However, HEOs have the potential to exist in a single-phase state, with their lattice inevitably experiencing local distortions due to variations in the atomic radii of cations and the presence of oxygen vacancies. These distortions are a consequence of the diverse array of cations present in equimolar or near-equimolar ratios within the lattice. The structural characteristics of HEOs are subject to a delicate balance between competing influences on the system’s free energy. On one hand, the high entropy composition tends to lower the free energy, promoting structural stability. Conversely, lattice distortion tends to increase the free energy by disrupting the ideal crystal lattice. This interplay between entropy-driven stability and lattice distortion underscores the tunability of HEO structures. External parameters, such as pressure, offer avenues to manipulate this equilibrium, thereby modulating the material’s structural properties. This intrinsic flexibility holds promise for tailoring HEOs to fulfill diverse application requirements. Further, a large positive Δ*H*_*mix*_ of metal oxides cannot be compensated by the HE effects, nor are their large negative values of Δ*H*_*mix*_ favorable for the creation of an entropy-driven single-phase mixed oxide. In the realm of electrocatalysis, the presence of lattice distortion and stacking fault defects induces a potent synergistic impact on the electronic structure of the catalyst, thereby endowing it with remarkable electrochemical activity. Owing to the limited number of synthesis reports and low statistical analysis of the database, the field of HEOs is still in its embryonic stage in comparison to the well-established field of HEAs.

### Cocktail Effect

The term “cocktail effect” has been employed as a comprehensive expression to characterize the emergent properties of HEMs that cannot be solely attributed to their individual components. For example, when predominantly light elements compose the HEOs, the overall density decreases. However, with the introduction of a metallic element with robust oxidative resistance, remarkable antioxidant activity is observed. These synergies extend to various aspects including thermoelectric response, mechanical properties, and catalytic and magnetic behaviors. Such phenomena are especially pronounced in catalysis, where the haphazard elemental distribution in the lattice generates a multitude of potential properties as functional materials in energy conversion and storage. The precise mechanisms responsible for the observed “cocktail effects” remain an unanswered question. However, in most instances, it is evident that increased stability and improved catalytic performance are conferred upon phases with higher levels of configurational disorder. The enhancement of the electrocatalytic activity of HEOs primarily arises from the creation of oxygen vacancies, synergistic interactions between multiple elements, and alterations in the adsorption energy of intermediates during reactions. Additionally, recent investigations have demonstrated that the lattice oxygen mediated mechanism can circumvent the conventional positive correlation between adsorption energies of oxygen-containing intermediates and catalytic performance in the oxygen evolution reaction (OER) process. This phenomenon has been corroborated in perovskite catalyst systems incorporating heteroatom doping.^46^ In principle, catalytic activity hinges on the characteristics of both the individual site and the surrounding elements.

## RECENT INNOVATIONS IN THE SYNTHESIS AND CRYSTAL STRUCTURE

**Figure 2:**
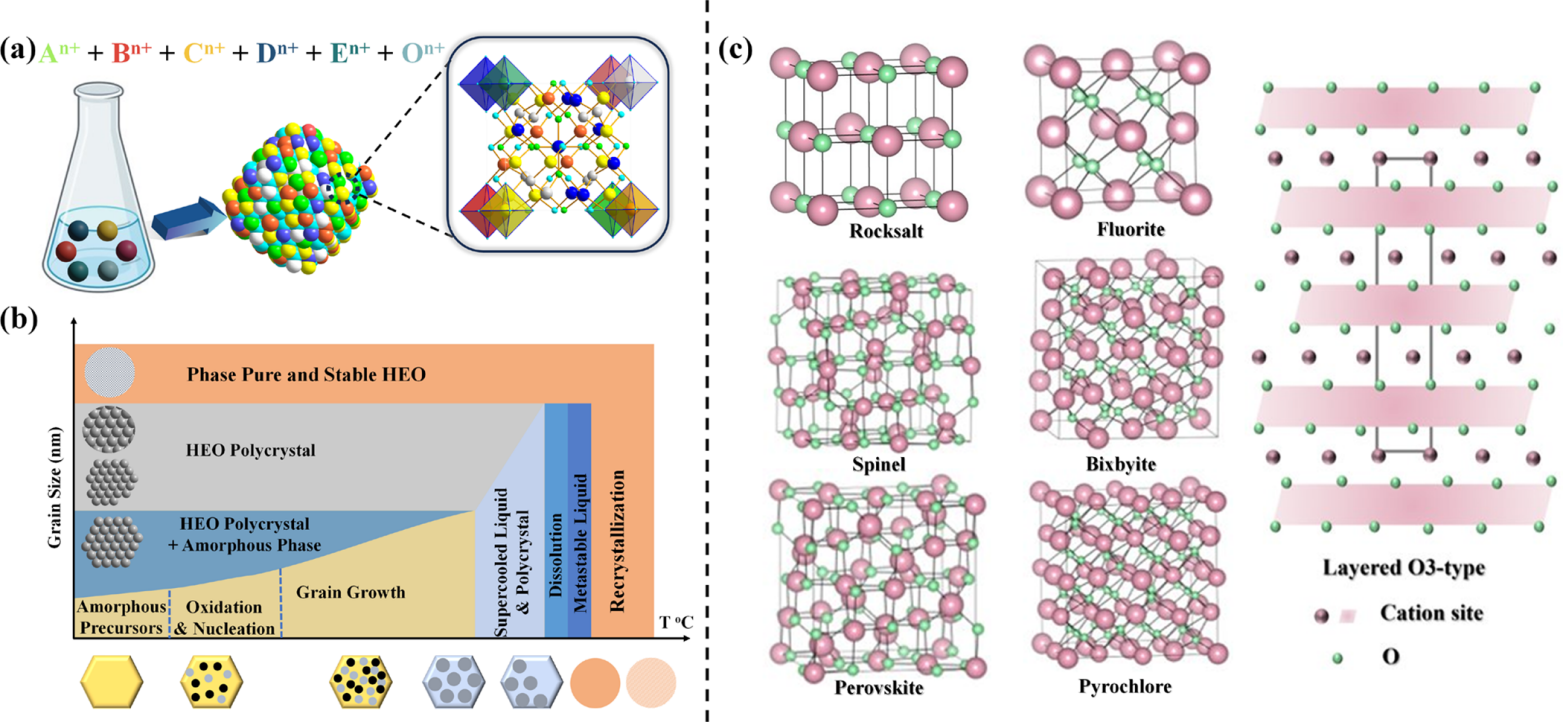
A general scheme depicting the (a) synthesis of HEO NPs and (b) their formation mechanism and recrystallization under different reaction conditions. (c) Commonly observed crystal structures of HEOs synthesized by various reaction pathways.

In 2015, Rost’s group reported the first HEO, Mg_0.20_Ni_0.20_Co_0.20_Cu_0.20_Zn_0.20_O, with a rock salt (RS) structure.^38^ Through rigorous experimentation with a simple thermodynamic model and a 5-component oxide system, they confirmed that entropy predominantly dictates the thermodynamic landscape. It guides a reversible solid-state transformation from a multiphase to a single-phase structure. The next major step toward reliably predicting the thermodynamic, kinetic, electronic, and phonon properties of HEOs involved constructing the appropriate atomic structure. Hence it became essential to tackle the inherent challenges such as simultaneous existence of intermediate compounds and byproducts. Such complexity posed challenges in identifying phases and comprehending reaction pathways essential for designing desired compounds and enhancing synthesis methods. Figure 2a–b shows a general schematic representation depicting the synthetic design of HEOs. Till date, the HEOs have been found to be mostly existing in six different crystal structures, viz. spinel, fluorite, pyrochlore, perovskite, bixbyite, and layered O3-type crystal structures (Figure 2c). Prior to delving into the pivotal factors influencing phase composition or properties within HEOs, it is essential to first briefly explore the synthesis methodologies and potential crystal structures associated with HEOs. This section aims to briefly shed light on the diverse synthesis procedures employed to obtain different HEOs and their derivatives, setting the stage for a deeper understanding of their properties and behaviors.

We believe that the growing interest in HEOs can be attributed significantly to their convenient and adaptable synthesis methods. However, it is worth noting that HEOs generally demand elevated synthesis temperatures, which hinders the efficient screening of their properties and poses a notable challenge in achieving nanoparticulate morphology during fabrication. Since their first report, several methods have been adopted for the synthesis of HEOs, and in this current Focus Review, we will discuss some of the newest oxide systems reported by different groups (from 2021 to early 2024) that enriched the traditional paradigm of HEOs by incorporating innovative synthesis methods, leading to enhanced control over material properties and opening new platforms for research and development.

Synthesis of HEOs faces constraints due to the challenge of incorporating or dissolving diverse elements with distinct properties into a single oxide structure, thus limiting their elemental combinations that can be utilized.^47^ Approaches such as the carbothermal shock (CTS) method utilize rapid heating and cooling of metal reagents supported on oxygenated carbon to synthesize HEOs.^48^ While the combination of high temperatures and the catalytic properties of liquid metals initiate swift particle “fission” and “fusion” processes, leading to the creation of homogeneous blends containing multiple elements, the rapid cooling enables kinetic regulation over thermodynamic mixing phases, allowing for the formation of crystalline SS NPs. Similarly, spray pyrolysis offers promise in synthesizing nanocrystalline HEOs by virtue of their brief exposure to high temperatures, minimizing potential issues related to prolonged heat exposure.^49^ Nonetheless, despite these short residence times, aggregation of crystallites persists, resulting in the formation of micrometer-sized particles. To mitigate such agglomeration challenges, alternative synthesis methods employing mechanochemistry,^50^ sonochemistry,^51^ or microwave assisted^52^ pathways have been devised. These techniques operate at lower temperatures, presenting a viable strategy to address aggregation concerns while facilitating the production of finely dispersed HEO NPs. Again, there are several emerging strategies for the synthesis of HEOs by colloidal chemistry. A prevalent approach entails introducing a solution containing a blend of metal precursors into a heated solvent, that also act as a reducing agent, simultaneously.^53^ As elevated temperatures are generally necessary for entropy stabilization, this makes it difficult, though not impossible, to synthesize colloidal HEOs due to variations in the reduction rates of cationic precursors^54−58^ and lack of comprehensive understanding of reaction intermediates.^59,60^ Also, in contrast to chalcogenides^61,62^ and phosphides,^63^ no universally recognized molecular precursors exist that decompose to liberate oxide anions in the reaction mixture, impeding the straightforward formation of oxides. Instead, the introduction of oxygen into oxides typically involves the use of reagents and solvents containing hydroxyl or alkoxy groups. Nevertheless, there are a few reports of HEO NPs synthesized via colloidal chemistry, where multiphasic NPs are prepared at lower temperatures and subsequently annealed at higher temperature to convert them into single-phase HEOs. Alternate approaches entail the thermal or catalyzed decomposition in solution of a complex containing M-O, such as metal alkoxides, stearates, or oleates, and/or the use of mild pressure in hydrothermal/solvothermal reactors. Presently, there are many other synthesis and processing pathways available for HEOs. Table 1 outlines the various synthesis methods, compositions, and applications of HEOs that have been reported in the recent past.

**Table 1:** Synthesis, Composition, Crystal Phase, and Application of Different HEOs

**Composition**	**Synthesis Method**	**Application**	**Ref**
(FeCoNiCrMn)_3_O_4_	Dealloying	Supercapacitors	(65)
(La_*x*_K_0.4–*x*_Ca_0.2_Sr_0.2_Ba_0.2_)-TiO_3+δ_	Solid-state sintering	Optoelectronics	(66)
Na_0.83_Li_0.1_Ni_0.25_Co_0.2_Mn_0.15_Ti_0.15_Sn_0.15_O_2−δ_	Solid-state reaction (SSR)	Na-ion Battery	(67)
BaZr_0.2_Sn_0.2_Ti_0.2_Hf_0.2_Ce_0.2_O_3−δ_, BaZr_0.2_Sn_0.2_Ti_0.2_Hf_0.2_-Y_0.2_O_3−δ_, BaZr_1/7_Sn_1/7_Ti_1/7_Hf_1/7_Ce_1/7_Nb_1/7_Y_1/7_O_3−δ_	SSR	Switch from dry to wet atmosphere	(72)
AlNiCoRuMoO	Dealloying	Zn-Air Batteries	(73)
Mg_0.2_Co_0.2_Ni_0.2_Cu_0.2_Zn_0.2_°	Sol–gel	CO oxidation	(74)
(CoNiMnZnFe)_3_O_3.2_	Mechanical alloying	Electrocatalysis	(75)
BaSn_0.16_Zr_0.24_Ce_0.35_Y_0.1_Yb_0.1_Dy_0.05_O_3−δ_	Sol–gel method	Protonic ceramic fuel cells	(77)
(CoCrFeMnNi)_3_O_4_	Reverse Coprecipitation	Electrocatalysis	(79)
Li-(FeCoNiCuZn)O	Wet-soaking treatment	Electrocatalysis	(81)
[Fe_0.35_Co_0.33_Zn_0.32_][Mn_0.71_Fe_0.90_Co_0.03_Ni_0.36_]O_4_	Supercritical hydrothermal process	Electrocatalysis	(39)
(La_0.2_Nd_0.2_Sm_0.2_Ca_0.2_Sr_0.2_)MnO_3_	Sol–gel method	Fuel cell cathode material	(84)
(Yb_0.2_Y_0.2_Lu_0.2_Sc_0.2_Gd_0.2_)_2_Si_2_O_7_	Sol–gel method	Corrosion resistant	(86)
(Y_0.2_Nd_0.2_Sm_0.2_Eu_0.2_Er_0.2_)AlO_3_	Coprecipitation method	Thermal conductive	(87)
La_2_(Zr_0.2_Ce_0.2_Hf_0.2_Sn_0.2_Ti_0.2_)_2_O_7_	Plasma spraying	Thermal barrier	(88)
(La_0.2_Nd_0.2_Sm_0.2_Eu_0.2_Gd_0.2_)_2_Zr_2_O_7_	SSR	Thermal barrier	(89)
(Na_0.2_Bi_0.2_Ba_0.2_Sr_0.2_Ca_0.2_)TiO_3_	SSR	Energy storage	(90)
(Mg_0.2_Co_0.2_Ni_0.2_Cu_0.2_Zn_0.2_)O	SSR	Anode for Li-ion batteries	(91)
(Bi_0.2_Na_0.2_K_0.2_La_0.2_Sr_0.2_)TiO_3_	Citrate acid method	Electrical energy storage	(92)
Li_*x*_(CoCrFeMnNi)_3–*x*_O_4–*y*_	Sol–gel method	Solid-sate electrolyte	(93)
(Mg_0.2_Co_0.2_Ni_0.2_Cu_0.2_Zn_0.2_)O	Joule heating	Anode for Li batteries	(94)
(Li_7_La_3_Al_0.5_Ti_0.5_V_0.5_Zr_0.5_)O_12_	SSR	Li-S battery	(46)
Sn_0.2_La_0.2_Pr_0.2_Sm_0.2_Y_0.2_)O_2−δ_, (Tb_0.2_La_0.2_Pr_0.2_Sm_0.2_Y_0.2_)O_2−δ_, (Zr_0.2_La_0.2_Pr_0.2_Sm_0.2_Y_0.2_)O_2−δ_, (Zr_0.2_La_0.2_Tb_0.2_Sm_0.2_Y_0.2_)O_2−δ,_ (Zr_0.25_La_0.25_Sm_0.25_Y_0.25_)O_2-δ_	SSR	Optoelectronics	(95)
La_0.2_Eu_0.2_Gd_0.2_Y_0.2_Yb_0.2_)_2_Zr_2_O_7_	Ball-milling followed by sintering	Single-phase forming ability	(96)
Ba(XTiZrTa)O_3_ X = (FeNb, MnNb, FeSn, FeV)	Ionic liquid-hydroxide-mediated technique	Low temperature synthesis	(97)
(GdLaNdSm)_1–*x*_Sr_*x*_MnO_3_	Spray pyrolysis method	Ingredient design	(49)
(Sm_0.19_Yb_0.19_Gd_0.19_Er_0.19_Dy_0.19_U_0.05_)_2_Ti_2_O_7_	SSR	Nuclear applications	(98)
(Ca_0.25_Sr_0.25_Ba_0.25_Pb_0.25_)Bi_2_Nb_2_O_9_, (Ca_0.2_Sr_0.2_Ba_0.2_Pb_0.2_Nd_0.1_Na_0.1_)Bi_2_Nb_2_O_9_	SSR	Relaxor ferroelectric materials	(99)
Li(Gd_0.2_Ho_0.2_Er_0.2_Yb_0.2_Lu_0.2_)GeO_4_	SSR	Microwave dielectric ceramic	(100)
(Eu_0.2_Bi_0.2_Y_0.2_La_0.2_Cr_0.2_)_2_O_3_/PVDF–PMMA	Electrospinning	Dielectric materials	(101)
CoMnFeZnYO_7_	Wet ball-milling followed by calcination	Thermistor material	(102)
(Sm_0.2_Eu_0.2_Gd_0.2_Tm_0.2_Yb_0.2_)_2_Zr_2_O_7_	SSR	Low-frequency sound absorption	(103)
(Co_0.2_Cr_0.2_Fe_0.2_Mn_0.2_Ni_0.2_)O	Combustion method	Dielectric materials	(104)
Ag(CuZn)(AlCr)_2_O_4_	Sol–gel combustion method	Photocatalyst	(105)
(Mg_0.2_Co_0.2_Ni_0.2_Cu_0.2_Zn_0.2_)O	SSR	Li-ion battery	(106)
(NiCoMnCuZn)WO_4_	Electrospinning	Li-S battery	(107)
FeCoNiCrMnO_*x*_	Sol–gel method	OER	(108)
(CeLaPrSmY)O_2–*y*_, Ce_0.8_(LaMnNdZr)_0.2_O_2–*y*_, Ce_*x*_(LaNdPrSm)_1–*x*_O_2–*y*_, Ce_*x*_(FeLaNdZr)_1–*x*_O_2–*y*_, Ce_*x*_(AlPrYZr)_1–*x*_O_2–*y*_	Sol–gel method	CO Oxidation	(109)

### Distinctive Heating and Quenching Techniques

**Figure 3:**
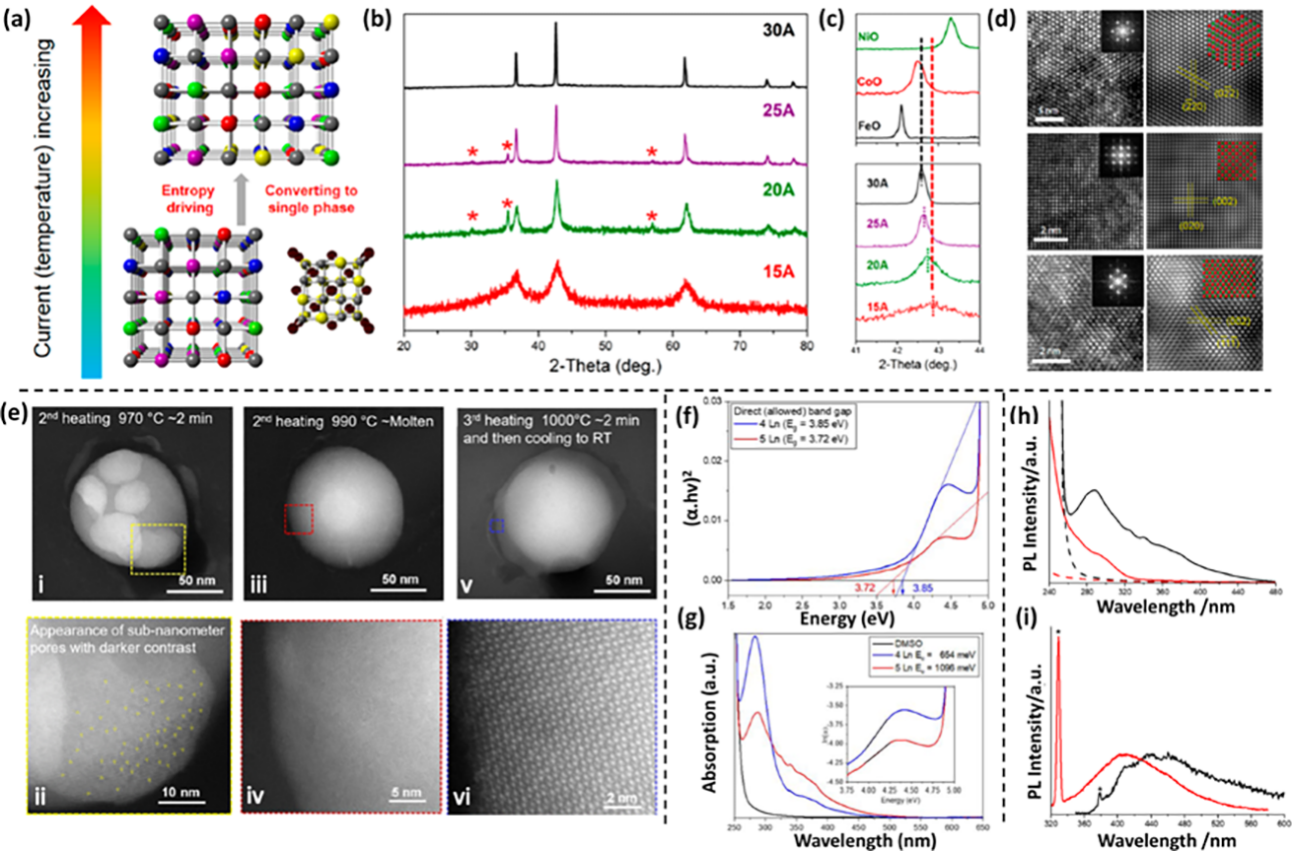
(a) Crystallographic representation of single-phase RS oxide formation. (b) XRD pattern showcasing the (MgFeCoNiZn)(OH)_2_ precursor subjected to Joule heating with varying currents. (c) Partial XRD pattern displaying the (MgFeCoNiZn)(OH)_2_ under different Joule-heating currents in comparison to parent RS oxides. (d) HRTEM micrographs captured along the [100], [111], and [110] axes and corresponding IFFT images and Selected Area Electron Diffraction (SAED) patterns. Panels (a–d) are adapted and reproduced with permission from ref (80). Copyright 2022 American Chemical Society. (e) HAADF-scanning transmission electron microscopy (STEM) micrographs deciphering (i–ii) Dissolution of small grains and the subsequent recrystallization of (La_0.2_Er_0.2_Sm_0.2_Yb_0.2_Y_0.2_)_2_Ce_2_O_7_. (iii) Formation of a liquid sphere at 990 °C. (iv) Marked area in (iii) revealing the presence of liquid phase. (v) During the final heating at 1000 °C the system exhibits notable thermal stability. (vi) Atomic-resolution HAADF STEM micrographs of the marked area in (v), revealing the lattice structure of the single-crystal HEFO oriented in the [111] direction. Panel (e) is reprinted with permission from ref (110). Copyright 2022 American Chemical Society. (f) Optical absorption spectra of samples containing 4 and 5 lanthanide (Ln) ions. (Inset: Urbach plot.) (g) Tauc plot manifesting the band gap energies (E_g_). Panels (f–g) are reprinted with permission from ref (85). Copyright 2021 American Chemical Society. (h–i) Comparative measurement of the absorption and photoluminescence (PL) for the bulk HE phases in their previous work (black) and for the HEMs in their current study (red). Panels (h–i) are reprinted with permission from ref (76). Copyright 2022 American Chemical Society.

The significant variation in ionic radius poses a considerable challenge in achieving single-phase HEOs due to the ionic size effect within the crystal structure. Additionally, at elevated temperatures, competing phase transitions, such as those between RS and spinel structures, further complicate the synthesis process, exacerbating the difficulty in obtaining single-phase HEOs. By employing Joule heating and exercising precise control over precursor synthesis, Hu and their research team accomplished the synthesis of three different HEOs (RS, spinel, and perovskite).^80^ Within a mere span of seconds, the Joule heating to nickel foil initiated the thermal decomposition of the precursor, facilitating the concurrent formation of RS-HEO with a novel composition of (MgFeCoNiZn)O. This process introduced Fe^2+^ ions into the RS structure, thus facilitating the evolution of the desired compound. Figure 3a presents a schematic diagram reaction mechanism of (MgFeCoNiZn)O formation, in which, at elevated synthesis temperatures, the RS structure transforms into a single-phase configuration driven by entropy. As the synthesis temperature gradually rises, the hydroxide precursor undergoes a thermal decomposition process, transitioning into two distinct structures—RS and spinel—and finally transforms into phase pure RS structure at a higher temperature. They conducted a detailed investigation into the formation mechanism of RS (MgFeCoNiZn)O using XRD, XPS, EXAFS, and TEM. In accordance with traditional crystallographic theory, lattice parameters typically exhibit a positive correlation with ionic radius. In the context of (MgFeCoNiZn)O, the average ionic radius of the metallic elements is observed to be 73.5 pm. This value is slightly lower than that of Co^2+^ and higher than that of Ni^2+^. Consequently, it suggests that the lattice parameters (*a* = *b* = *c*) of (MgFeCoNiZn)O would likely be slightly smaller than those of CoO. Notably, the XRD peak (200) of the synthesized nanomaterial (Figure 3b–c) at 15 A lies between those of CoO and NiO, while for the sample synthesized at 30 A, it slightly surpasses that of CoO. This suggests that Fe^2+^, possessing the largest ionic radius, is not entirely incorporated into the RS structure at 15 A.

The HRTEM images in Figure 3d, along with their corresponding inverse FFT images in the insets, depict the atomic arrangement along the three fundamental axes of the RS structure. Also, the synthesized RS-HEOs featured significant advancement in promoting OER activity. Furthermore, the achievement of synthesizing spinel and perovskite HEOs by this group outlined the broad applicability of Joule heating utilizing nickel foil in the synthesis of HEOs.

The atomic-scale nucleation and growth mechanisms of HEOs are essential for effectively designing their structure and functionalities. To explore the atomistic information governing the formation of HEOs, Gao and colleagues prepared a polymeric precursor for HE (La_0.2_Er_0.2_Sm_0.2_Yb_0.2_Y_0.2_)_2_Ce_2_O_7_ in the fluorite oxide phase (HEFO) using the sol–gel process.^110^ They investigated the morphology and elemental transformation throughout its formation, primarily using atomic resolution in situ gas-phase STEM. The formation process unfolds in four distinct stages: first, nucleation arises during the oxidation of the polymeric precursor (pyrolysis) below 400 °C, where numerous nanoscale pores are observed. These pores facilitate gaseous oxygen diffusion and expedite the oxidation of the metal components, thereby promoting nucleation. Second, the diffusive grain growth occurs below 900 °C, facilitating structural development. Third, liquid-phase-assisted homogenization of the composition transpires under a “state of supercooling” at 900 °C. During this phase, most of the nanoscale pores diminish, leading to a gradual reduction in nanoparticle volume. Additionally, the size and morphology of the nanograins become discernible. Upon reaching temperatures exceeding 900 °C, the NPs underwent a transition back into a liquid phase. Detailed in situ STEM images recording the formation of the liquid phase are shown in Figure 3e (i–iv). The melting of oxide nanoparticles at elevated temperatures not only occurred at the interface between liquid and solid phases but also led to the formation of small pores within the grains, as indicated by the yellow arrow heads in Figure 3e (ii). Consequently, the grains in HEFO experienced dissolution at various scattered locations. Finally, entropy-driven recrystallization and stabilization manifest at elevated temperatures, completing the transformation process. The formation of the larger nanocrystal (NC) is thermodynamically stable both during cooling and upon reheating at 1000 °C (as observed in the third heating process and depicted in Figure 3e (v, vi). Based on all these advanced characterizations, they revealed the growth mechanism of the NCs at the atomic scale with the help of a schematic like in Figure 2b. Such formation mechanisms hold significant importance for the future design and synthesis of HEFO and other HEO materials with precise control over their morphology, structure, and resulting properties.

Utilizing synthetic pathways that incorporate molecular precursors offers several notable advantages compared to traditional methods of sample preparation. These precursors encompass nearly all atoms present in the desired product in proximity. During synthesis they undergo molecular-level mixing before decomposition, purification, and thorough characterization. Lewis and co-workers employed lanthanide dithiocarbamates as adaptable starting materials that are capable of being combined at the molecular level before undergoing controlled heating to generate HE lanthanide oxysulfide (Pr_0.51_Nd_0.51_Gd_0.50_Dy_0.48_SO_2_ (4 Ln) and Pr_0.42_Nd_0.41_Gd_0.42_Dy_0.42_Er_0.33_SO_2_ (5 Ln)) compounds.^85^ They analyzed the optical characteristics of the materials by observing the absorption spectra of both samples in dimethyl sulfoxide. The absorption spectra reveal a notable increase only in wavelengths shorter than 300 nm. Specifically, the samples exhibit absorption onsets around 515 and 550 nm for the 4 Ln and 5 Ln samples, respectively. In both cases, absorption intensifies at shorter wavelengths, exhibiting a distinct peak around 280 nm. Notably, the 4 Ln sample displays a more pronounced feature at this wavelength, while the 5 Ln sample demonstrates relatively elevated absorption across longer wavelengths (375–550 nm). The *E*_g_ and Urbach energy (*E*_u_) values were determined from the absorption spectra through Tauc plot and Urbach analysis methods. In their analysis, they examined scenarios of both direct and indirect bandgap transitions and found only a slight disparity (approximately 300 meV) between the obtained values. Therefore, the reported values are an average derived from extrapolating the fitted linear regions of the curves. The higher *E*_u_ observed for the 5 Ln material aligns with the anticipated greater disorder resulting from a higher number of elements on the metal sublattice.

Soon after this, they reported the first quantum confined lanthanide oxysulfide system as the host phase for an equimolar mixture of Nd, Pr, Gd, Dy, and Er.^76^ In comparison to the bulk sample of identical composition, the dispersion of these colloidal NPs exhibited a notable blue shift in both absorption and PL spectra. The absorption edge occurred at 330 nm with a peak wavelength (λ_max_) of 410 nm, in contrast to the bulk sample, where the absorption edge was observed at 500 nm with a λ_max_ of 450 nm. This pronounced shift suggests quantum confinement effects are at play. To discern whether the observed phenomenon is attributed to surface characteristics or strain-induced effects, they performed DFT calculations. They utilized DFT to predict the alteration in bandgap for the individual lanthanide oxysulfide end points under varying compressions and expansions around their equilibrium volumes, encompassing strains ranging from −5% to +5%. In brief, the calculations indicated that neither strain nor surface effects exhibit the magnitude necessary to explain the observed blue-shifts in the experimental optical spectra. Instead, these results further suggested that quantum confinement, stemming from the small lateral dimensions of the NCs (<10 nm), is possibly the primary factor driving these spectral shifts.

### High-Throughput Characterization

**Figure 4:**
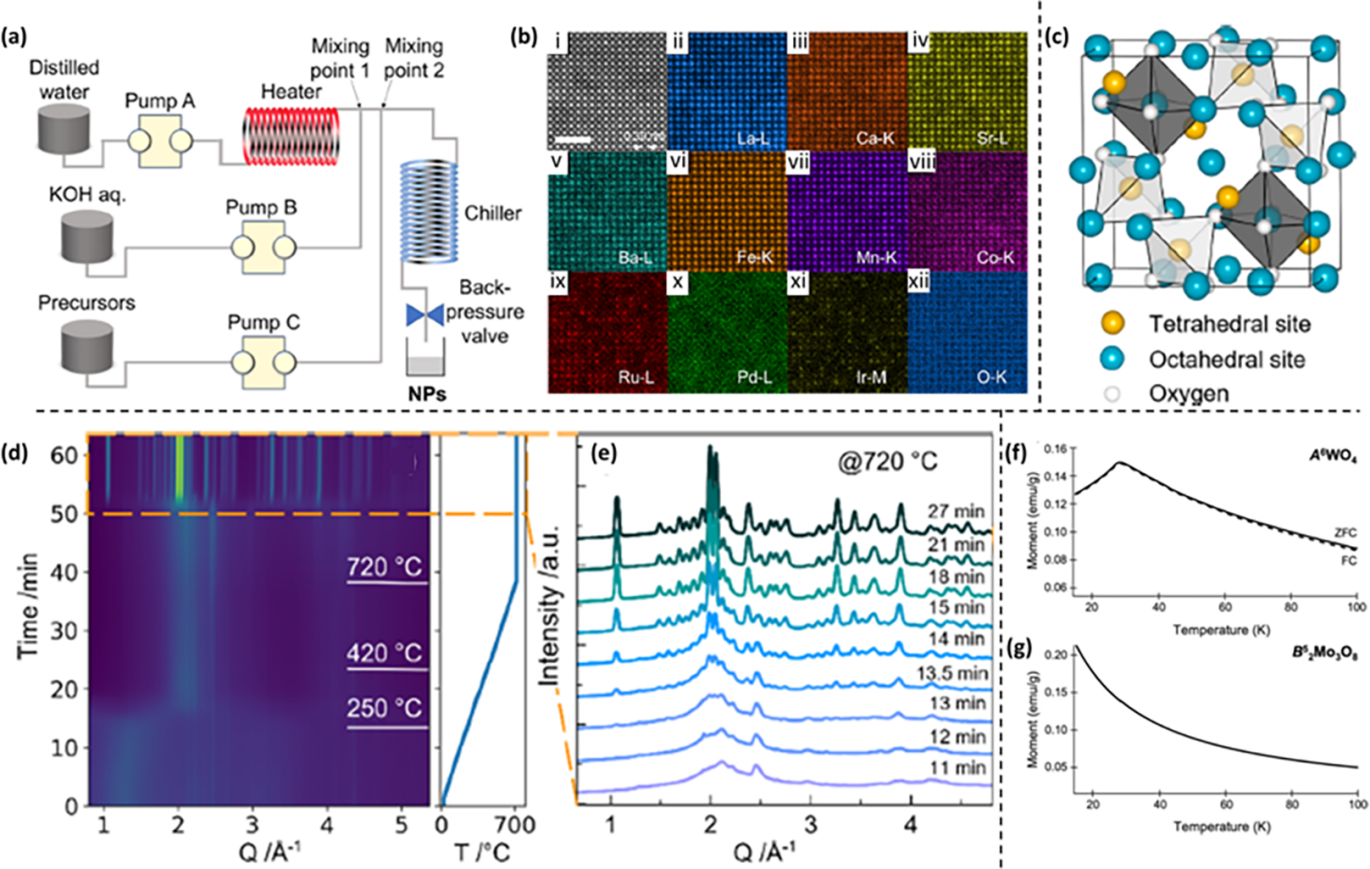
(a) Schematic layout of the flow reactor. (b) HAADF-STEM micrograph and EDS maps (i–xii) of HEO10 NPs (NPs). The scale bar represents 2 nm. Panels (a–b) are reprinted with permission from ref (111). Copyright 2024 American Chemical Society. (c) Cubic spinel structure comprising tetrahedrally coordinated (yellow) and octahedrally coordinated (blue) cation sites. Panel (c) is reprinted with permission from ref (112). Copyright 2022 American Chemical Society. (d) Temperature-dependent XRD patterns from room temperature to 720 °C and temperature profile. (e) Selected XRD collected at 720 °C. Panels (d–e) are reprinted with permission from ref (113). Copyright 2023 American Chemical Society. Magnetization data reveals that (f) A^6^WO_4_ exhibits AFM behavior with a TN of 30 K, while (g) B_2_^5^ Mo_3_O_8_ displays paramagnetic properties. Panels (f–g) are adapted with permission from ref (114). Copyright 2023 American Chemical Society.

Using a continuous supercritical hydrothermal flow-synthesis method, Kitagawa and co-workers developed denary HEO perovskite (HEO10) nanocubes at 450 °C and 30 MPa within 1 s.^111^Figure 4a depicts the diagrammatic representation of the flow reactor employed in their experiment. The precursor solution was formulated by dissolving all ten cationic precursors and HNO_3_ in deionized water. The addition of HNO_3_ served the purpose of preventing the reduction of Pd by lowering the pH. Subsequently, a 0.6 M KOH aqueous solution and a metal precursor solution were both pumped, where KOH solution combined with the supercritical water at mixing point 1, followed by the injection of the metal reagent solution at mixing point 2. The elemental distribution of the HEO10 nanocubes was analyzed by recording atomic-resolution HAADF-STEM EDS mapping, as depicted in Figure 4b (i–xii). The plane distance along the ⟨100⟩ direction, 0.39 nm, was in consonance with the findings from XRD. The EDS maps revealed a uniform dispersion of all elements throughout the sample. The HEO nanocubes displayed superior catalytic performance for the CO oxidation reaction compared to the LaFeO_3_ NPs.

Hallas and co-workers synthesized polycrystalline spinel HEO (Cr, Mn, Fe, Co, Ni)_3_O_4_ and its gallium-substituted analogs (with 10%, 20%, and 40% Ga) through a solid-state reaction method.^112^ The cubic spinel structure (AB_2_O_4_), illustrated in Figure 4c, consists of two distinct cation sublattices. While the first sublattice, depicted in yellow, features tetrahedral coordination denoted as the A sublattice, the second sublattice, highlighted in blue, showcases octahedral coordination known as the B sublattice. These A and B sublattices independently form diamond and pyrochlore networks, respectively. Drawing parallels with other known spinels based on 3d transition metals (TMs), one might expect a pronounced preference for specific sites among the various cations. However, the similarity in atomic form factors among the elements involved makes it challenging to resolve this preference using XRD. The refined lattice parameters and adjustable oxygen coordinated as a function of Ga substitution. Because of its remarkable site selectivity, the magnetic ground state of the spinel HEO can be significantly altered by substituting nonmagnetic Ga in concentrations ranging from 0% to 40%. Through magnetic susceptibility and powder neutron diffraction measurements, they observed that ferrimagnetic order remains stable within this range. X-ray absorption and magnetic circular dichroism studies have revealed that the introduction of Ga involved valence and site reorganizations specifically affecting Mn, Fe, and Co, whereas Ni and Cr remain unaffected. For the 20% Ga sample, an exceptionally high-entropy system with *S*_*config,total*_ exceeding 3*R* was achieved. Furthermore, they demonstrated that by selecting appropriate cations, the individual sublattice entropies can be finely tuned and engineered.

The existent HEOs commonly adopt the RS and spinel crystal structures. This preference arises from the availability of numerous elements capable of adopting these structures. Hence, it is crucial to synthesize and characterize a broader range of HEO materials encompassing various families of crystal structures. This endeavor is significant due to the pivotal roles that composition and crystal structure play in determining band structure and properties. Considering this situation, Jansen et al. investigated the feasibility and challenges associated with synthesizing mullite-type hexagonal HEOs^113^ and synthesized five prototype compositions, namely Bi_2_M_4_O_9_ (where M = Ga^3+^, Al^3+^, and Fe^3+^) and four A_2_Mn_4_O_10_ (where A = Nd, Sm, Y, Er, Eu, Ce, and Bi), which possess intricate structures. In brief, they examined the potential routes and techniques for producing these materials in the laboratory. The formation process was monitored using in situ XRD and X-ray spectroscopy (XAS). Despite cocrystallization being common in this system, the in situ XRD observations revealed that all the materials followed a pathway involving an amorphous intermediate stage, with no evidence of crystalline phase formation, as illustrated in Figure 4d–e (Bi-HEO composition). The HEO formation occurs in two distinct stages. Initially, the amorphous precursor undergoes transformation into a second amorphous phase with a different structure around 300 °C. This intermediate amorphous phase then directly crystallizes into respective HEOs. The relatively lower temperature of the first transition suggests that the precursor powder likely contains constituents such as nitrates or organics, which typically decompose within this temperature range.

The transition of the amorphous precursor into the amorphous intermediate phase is marked by a significant shift in the main scattering position, from approximately ∼1.3 to ∼2.2 Å^–1^ (as depicted in Figure 4d). This increase in the scattering angle indicates transition of a larger phase into a smaller one, consistent with the decomposition of a phase due to the loss of its organic components. For Bi-HEOs, the disappearance of Bragg peaks from Ga_2_O_3_ coincided with the formation of the mullite-type structure. While formation of Bi-HEO required approximately 14 min of annealing at 720 °C, the Er-HEO, Eu-HEO, and Ce-HEO begin to crystallize at 670 °C. However, the first reflections of RE-HEO become visible after 22 min at 720 °C. But, even after 100 min at 720 °C, the intermediates do not entirely transform into mullite-type structure. No significant change was observed in the XAS spectra, indicating that the transitioning of the reagents into the amorphous intermediate sets the stage for the subsequent crystallization of the final products.

Schaak and co-workers reported the synthesis of two HEO systems by high-temperature solid-state combustion at 900 and 1100 °C, respectively.^114^ A HE tungsten oxide, denoted as A^6^WO_4_ (where A = Mn, Fe, Co, Ni, Cu, and Zn), exhibited a monoclinic wolframite crystal structure and displayed characteristics of a narrow bandgap antiferromagnet. On the other hand, B_2_^5^ Mo_3_O_8_ (where B = Mn, Fe, Co, Ni, and Zn) is a HE molybdenum oxide with a hexagonal crystal structure, functioning as a semimetallic paramagnet. A^6^WO_4_ demonstrated long-range antiferromagnetic (AFM) ordering with a transition temperature (TN) of 30 K (Figure 4f). This TN is notably 15 K lower than the average TN of 45.2 K observed in other AFM parent compounds. This finding suggests that despite the presence of competing magnetic interactions within the HEO phase, they can still attain long-range ordering at this temperature. Moreover, the presence of only one AFM transition indicates that the mixing of magnetic 3d TMs at the A site is uniform. This uniformity suggests that there is no phase segregation due to clustering of different 3d elements, which would otherwise lead to multiple magnetic transitions.

In contrast to tungstate parent compounds, most B_2_Mo_3_O_8_ molybdates exhibit a mix of AFM and ferromagnetic behaviors, except for Zn_2_Mo_3_O_8_, which is paramagnetic. However, the B_2_^5^ Mo_3_O_8_ HEO phase is found to be paramagnetic (Figure 4g). This observation suggested that the relatively low concentration of magnetic elements on the B sites, combined with competing magnetic correlations favored by the different metals, renders the exchange interaction-mediated magnetic ordering unstable. Consequently, long-range magnetic order is not sustained. In summary, the magnetic behavior indicated a single magnetic transition for A^6^WO_4_, while B_2_^5^Mo_3_O_8_ shows a lack of magnetic transitions. This supports the notion that there is no phase segregation, as the individual magnetic transitions of the parent phases would have been observed if they had segregated out.

### Innovative Surface Performance and Stability

**Figure 5:**
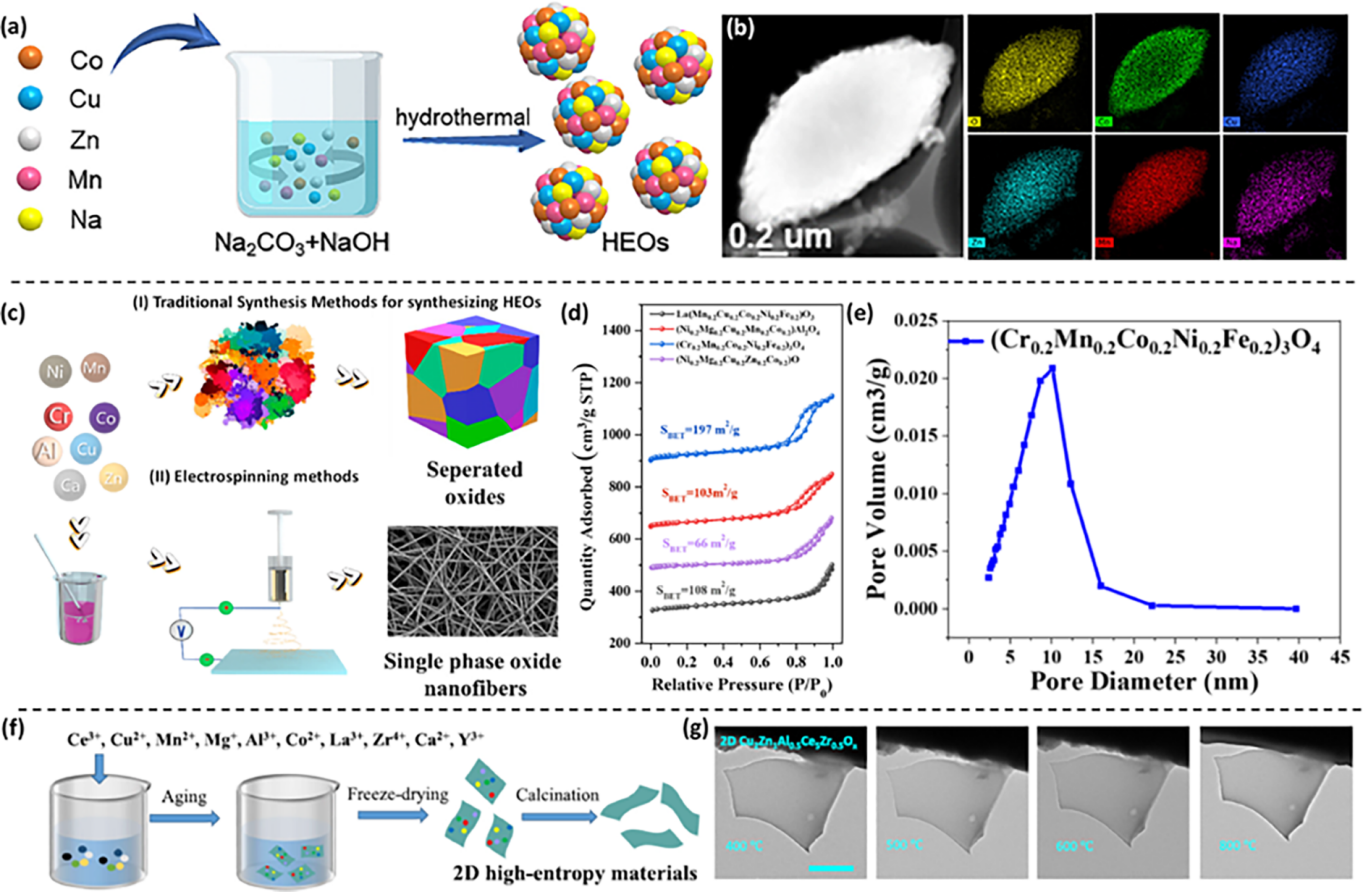
(a) Pictorial representation of the hydrothermal synthesis of (CoCuZnMnNa)O_*x*_ HEOs. (b) Elemental mapping images obtained through TEM-EDX showcase of the elemental distribution. Panels (a–b) are adapted with permission from ref (70). Copyright 2023 Royal Society of Chemistry. (c) Scheme depicting the synthesis of HEOs involving different methods, with (I) traditional approaches like the coprecipitation and solvothermal methods showing limitations and (II) electrospinning methods. (d) The texture characterizations of HEO nanofibers are elucidated through N_2_ sorption isotherm curves for various compositions: (Cr_0.2_Mn_0.2_Co_0.2_Ni_0.2_Fe_0.2_)_3_O_4_, (Ni_0.2_Mg_0.2_Cu_0.2_Mn_0.2_Co_0.2_)Al_2_O_4_, Ni_0.2_Mg_0.2_Cu_0.2_Zn_0.2_Co_0.2_O, La(Mn_0.2_Cu_0.2_Co_0.2_Ni_0.2_Fe_0.2_)O_3_. (e) Barrett–Joyce–Halenda (BJH) pore size distribution curves of (Cr_0.2_Mn_0.2_Co_0.2_Ni_0.2_Fe_0.2_)_3_O_4_. Panels (c–e) are adapted with permission from ref (115). Copyright 2024 American Chemical Society. (f) Schematic of the reaction mechanism of the synthesis of 2D HEO. (g) In-situ TEM images of the 2D Cu_2_Zn_1_Al_0.5_Ce_5_Zr_0.5_O_*x*_ captured at different temperatures of RWGS. Panels (f–g) are adapted with permission from ref (69). Copyright 2023 Springer Nature.

Recent experiments by various groups have demonstrated that HEOs also exhibit exceptional potential in organic photocatalysis.^78^ Inspired by such reports, Li et al. developed a simple hydrothermal synthesis approach to generate HEO (CoCuZnMnNa)O_*x*_ NPs for organic photocatalytic conversion.^70^Figure 5a illustrates the schematic of the hydrothermal process employed for the synthesis of the HEOs. For better understanding of the constituents within the HEOs, an EDX characterization was performed (depicted in Figure 5b). The diverse metals (Co, Cu, Zn, Mn, and Na) were distinctly observed to be evenly dispersed within the HEO NPs, showcasing the characteristic “dinosaur egg” morphology. Under mild conditions, these NPs were effectively activated by visible light, enabling the attainment of optimal yields and selectivity in sulfide oxidative coupling reactions and benzimidazole cyclization reactions across a broad spectrum of substrates.

Dai and co-workers demonstrated an electrospinning method to fabricate a wide range of one-dimensional HEO nanofibers, that include an inverse spinel (Cr_0.2_Mn_0.2_Co_0.2_Ni_0.2_Fe_0.2_)_3_O_4_, perovskite La-(Mn_0.2_Cu_0.2_Co_0.2_Ni_0.2_Fe_0.2_)O_3_, spinel (Ni_0.2_Mg_0.2_Cu_0.2_Mn_0.2_Co_0.2_)Al_2_O_4_, and cubic Ni_0.2_Mg_0.2_Cu_0.2_Zn_0.2_Co_0.2_O.^115^Figure 5c presents the synthesis illustration of the aforementioned materials by (I) traditional approaches like the coprecipitation and solvothermal method and (II) electrospinning methods consisting three steps. They observed that while the conventional synthesis pathways such as coprecipitation and solvothermal necessitated the requirement of a high temperature calcination step, to obtain single-phase HEOs, the elevated temperatures led to the structural breakdown of pores, giving rise to bulky HEOs on a micron scale with low surface areas. Throughout the electrospinning process, the metal precursors were evenly dispersed and exhibited a high degree of disorder. This favorable condition facilitated the crystallization into structurally disordered HE phases. The variance in configurational energy among various cation sites diminished, promoting their formation at a lower single-phase crystallization temperature. In Figure 5d, the data shows the Brunauer–Emmett–Teller (BET) isotherm curve, which is a measure of N_2_ sorption, of all the obtained HEO nanofibers. The specific surface areas of nanofibers composed of these HEOs fell within the range of 66–197 m^2^ g^–1^, which was much higher than the specific surface areas of HEOs prepared by other conventional methods. Also, the N_2_ adsorption of HEO nanofibers exhibited a gradual increase in uptake from a relative pressure of 0 to 0.6. However, a sudden surge in uptake was observed in the range of *P*/*P*_0_ = 0.6–0.9. In all cases, the sorption curves displayed type IV isotherms, indicative of a mesoporous structure, as depicted from the pore size distribution curves of (Cr_0.2_Mn_0.2_Co_0.2_Ni_0.2_Fe_0.2_)_3_O_4_ in Figure 5e. Such high surface area values of HEOs were attributed to the low single-phase crystallization temperatures, and the anticipated outcome of the nanoscale fiber morphology.

As Cu-based nanocatalysts serve as crucial components in numerous industrial catalytic processes, enhancing both the catalytic performance and stability of Cu-nanocatalysts represents a persistent challenge. In their recent work, Ye et al. adopted a polyvinylpyrrolidone (PVP) synthesis strategy and the HE principle to alter the structure of Cu-based 2D HEOs comprising six to eleven distinct elements,^69^ as presented in Figure 5f. The as-synthesized catalyst exhibited enhanced CO_2_ hydrogenation activity, achieving a pure CO production rate of 417.2 mmol g^–1^ h^–1^ at 500 °C. Remarkably, this rate is four times higher than that reported for advanced catalysts. To directly witness the structural changes of catalysts during the process of reverse water-gas shift (RWGS), they conducted in situ characterization using an environmental TEM setup. Specifically, they examined the pristine 2D Cu_2_Zn_1_Al_0.5_Ce_5_Zr_0.5_O_*x*_ catalyst. Remarkably, during the heating ramp from 400 to 800 °C, no sintering phenomenon was observed, as depicted in Figure 5g. The crystal structure of 2D Cu_2_Zn_1_Al_0.5_Ce_5_Zr_0.5_O_*x*_ remained robust even after exposure to 800 °C of RWGS, as confirmed by XRD analysis, HAADF-STEM images, and electron diffraction pattern examination. Thus, the HE 2D materials offer a novel pathway to achieve both catalytic activity and stability simultaneously.

**Figure 6:**
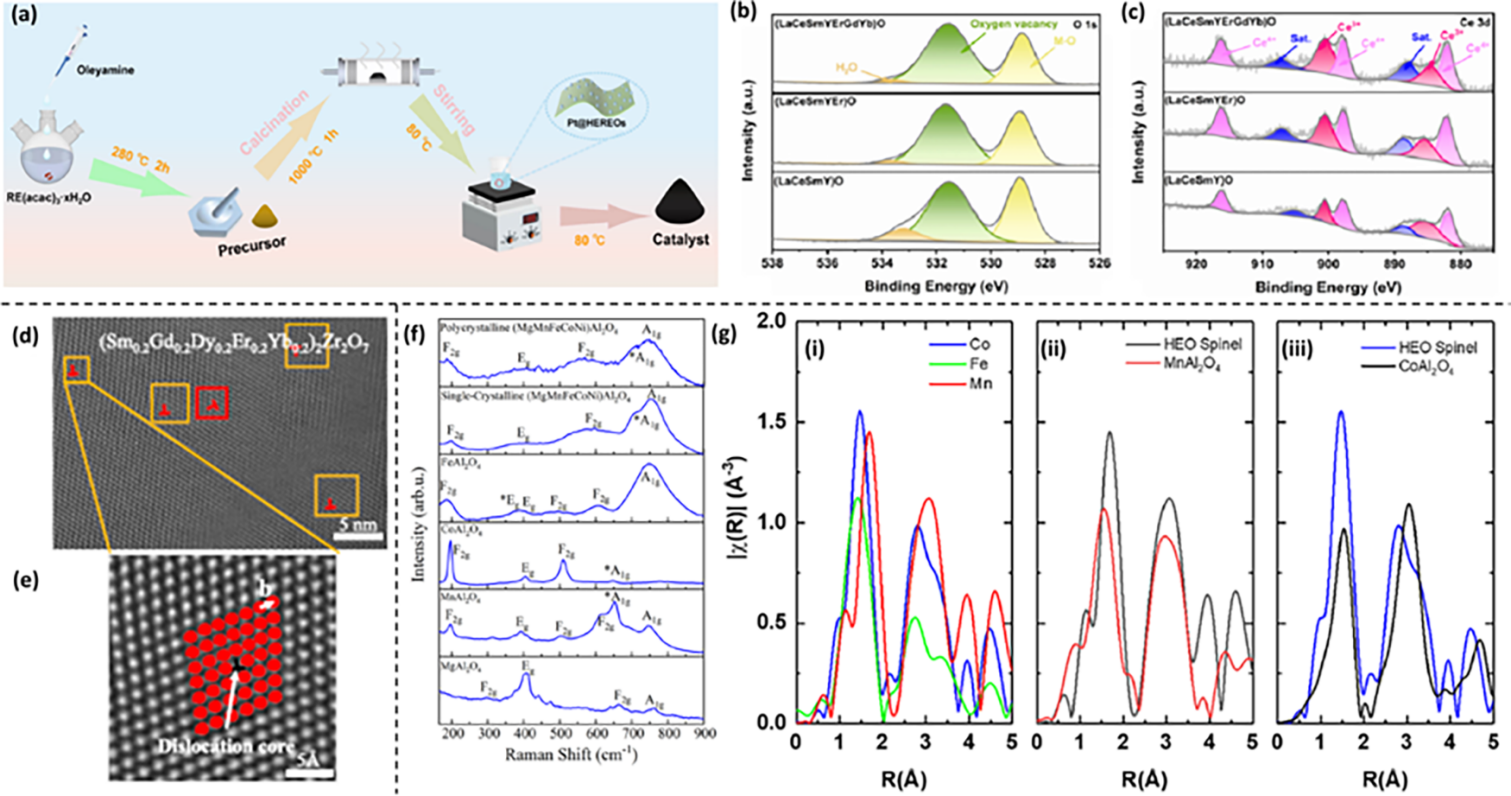
(a) Schematic representation of the reaction pathway for the synthesis of Pt-HEREOs. (b–c) High-resolution X-ray photoelectron spectroscopy (HR-XPS) O 1s spectra, and Ce 3d spectra of (LaCeSmY)O, (LaCeSmYEr)O, and (LaCeSmYErGdYb)O. Panels (a–c) are reprinted with permission from ref (116). Copyright 2024 American Chemical Society. (d) HRTEM image of HEFO. (e) The inverse FFT filtered image of HEFO. Panels (d–e) are reprinted or adapted with permission under a Creative Commons Attribution 4.0 International License from ref (117). Copyright 2022 Springer Nature. (f) Raman spectra for the polycrystalline and single crystalline (MgMnFeCoNi)Al_2_O_4_ samples and their parent compounds, MgAl_2_O_4_, MnAl_2_O_4_, FeAl_2_O_4_, and CoAl_2_O_4_. (g) Fourier transform (FT) of the k^2^χ(k) EXAFS spectra for (MgMnFeCoNi)Al_2_O_4_ and two parent compounds, MnAl_2_O_4_ and CoAl_2_O_4_. Panel (i) illustrates the comparison among Co, Fe, and Mn absorbers within the same HEO sample, showcasing variations in bond lengths and potential degrees of cation inversion. Panel (ii) contrasts the HEO-Mn data with that of MnAl_2_O_4_. In panel (iii), a comparison is made between HEO-Co and CoAl_2_O_4_. Panels (f–g) are reprinted or adapted with permission under a Creative Commons CC BY license from ref (118). Copyright 2023 American Institute of Physics.

HEOs possess the ability to incorporate various cationic sites within their structure, that offers significant flexibility in the formation of oxygen vacancies. This tunability holds promise for a wide range of applications, leveraging the unique properties of HEOs to enhance performance and functionality.^4^ Conversely, despite having poor durability and scarcity and being expensive, Pt is widely regarded as the most ideal material for hydrogen evolution reaction (HER). Taking this into consideration, Huang and Du recently reported the design of pH-universal and corrosion-resistant high entropy rare earth oxides (HEREOs) catalysts with Pt NPs anchored on the oxygen vacancy.^116^ The process to obtain the precursors for HEREOs involves mixing rare earth acetylacetone salt with oleylamine in equal proportions at 280 °C for 2 h. Subsequently, the HEREOs precursor undergoes annealing in a muffle furnace at 1000 °C for 1 h to produce the final HEREOs. Pt-HEREOs were subsequently synthesized through the deposition of PtCl_4_ onto the HEREOs employing an evaporative solvent technique. A schematic of the entire reaction pathway is illustrated in Figure 6a.

XPS was employed to unveil the surface chemical properties and electronic configurations of the HEREOs. The study aimed to investigate the correlation between oxygen vacancy and system entropy in selected compositions of rare earth oxides, namely (LaCeSmY)O, (LaCeSmYEr)O, and (LaCeSmYErGdYb)O, as shown in Figure 6b. In the HR-XPS spectrum of the O 1s peak, three distinct peaks were observed at binding energies of 528.9, 531.6, and 533.4 eV, respectively. These peaks signify characteristic features: the first peak represents the metal–oxygen (M–O) bond, the second peak indicates oxygen vacancies, and the third peak suggests the presence of adsorbed hydroxyl species or H_2_O. As the peak area ratio associated with oxygen vacancies rises from 48.3% in (LaCeSmY)O to 72% in (LaCeSmYErGdYb)O, it suggests a notable trend. This trend supports the conclusion that an increase in the entropy value of the system facilitates the formation of surface oxygen vacancies. The absence of noticeable shifts in the binding energies of (LaCeSmY)O, (LaCeSmYEr)O, and (LaCeSmYErGdYb)O in the Ce 3d spectrum with the increase in the configuration entropy of the system is indicative. It suggests that the high entropy effect of the system does not exert significant influence on the electronic structure of Ce (Figure 6c). Again, no discernible shift in the binding peak of the O 1s spectrum was observed following Pt loading and various temperature treatments. The decrease in oxygen vacancy concentration following Pt loading can be attributed to the anchoring of some Pt NPs onto the surface oxygen vacancies of (LaCeSmYErGdYb)O. This anchoring process enhances the activity and stability of the catalyst to a certain extent. Hence, HEREOs featuring abundant surface defects serve a dual purpose: they not only provide stability to the NPs deposited on the substrate surface but also exert a substantial influence on the electronic structure of these NPs.

Dislocation is prevalent and significant in metals, but their impacts are not well understood in oxide ceramics. This lack of recognition stems from the substantial strain energy associated with the rigid ionic/covalent bonding in these ceramics, resulting in dislocations with low density. Additionally, these dislocations exhibit thermodynamic instability and spatial inhomogeneity. Han et al. discovered ultrahigh-density edge dislocations in high-entropy fluorite oxide (HEFO).^117^ They analyzed the atomic microstructure of rare-earth zirconate (Gd_2_Zr_2_O_7_) and HEFO (Sm_0.2_Gd_0.2_Dy_0.2_Er_0.2_-Yb_0.2_)_2_Zr_2_O_7_ by the HRTEM along the [110] zone axis. The atomic structure of Gd_2_Zr_2_O_7_ was characterized by a pristine arrangement, devoid of lattice distortions or imperfections. As the composition complexity was increased, highly dense edge dislocations became apparent in the atomic image of HEFO, as depicted in Figure 6d. These dislocations within HEFO are distinctly observable within the highlighted yellow square area. Figure 6e illustrates an enlarged depiction of a single edge dislocation, specifically corresponding to the upper left edge dislocation in Figure 6d. This dislocation possesses a Burgers vector of 1/2[111]. In Figure 6e, rather than displaying uniform fringe patterns, edge dislocations with identical Burgers vectors are clearly observable. This observation aligns closely with the atomic image in real space. This is to note that there consistently exist two types of dislocations with distinct Burgers vectors. The prevalence of dislocations with Burger vectors of 1/2[111] surpasses those with other Burgers vectors. This predominance can be attributed to the smaller interplanar distance of planes and, consequently, the lower strain energy associated with this configuration. The dislocation density in HEFO was calculated to be approximately 10^9^ mm^–2^, significantly exceeding that of conventional oxide ceramics, and it even surpassed the values observed in certain metals or alloys. Hence, their research demonstrated a progressive and thermodynamic stabilization of dislocations with increasing complexity in composition. This stabilization occurs through entropy gains that can offset the strain energy associated with dislocations. Furthermore, they observed a significant enhancement of fracture toughness, approximately 70%, in pyrochlore ceramics featuring multiple valence cations. This enhancement was attributed to the interaction between cracks and the enlarged strain field surrounding immobile dislocations, leading to the deflection and bridging of cracks. This finding has the potential to advance our conception of the atypical properties exhibited by HE ceramics and expedite the discovery of novel features and applications within the field.

Single crystals offer a unique opportunity to delve deeper into the impact of lattice distortions on the crystal structure and physical characteristics of HEMs. Mao et al. are the first to report the single crystals of (MgMnFeCoNi)Al_2_O_4_ HEOs with the spinel structure, using the optical floating zone growth method.^118^ They utilized a mixed powder containing MgO, MnO, FeO, CoO, NiO, and Al_2_O_3_ in a stoichiometric ratio to fabricate feed and seed rods. Initially, the mixture of source materials was loaded into a cylindrical balloon. Subsequently, the balloon containing the source material was inserted into a quartz tube and subjected to isostatic pressing at 60 MPa. The seed rod was produced in a similar manner. Following preparation, the rods underwent sintering for 48 h at a temperature of 1200 °C. Finally, the optical floating zone growth technique was employed to obtain the desired single crystal. The obtained composition exhibited a cluster spin glass (CSG) phase. Figure 6f displays the Raman spectra of both single-crystalline and polycrystalline samples of HE (MgMnFeCoNi)Al_2_O_4_, alongside those of the parent compounds MgAl_2_O_4_, MnAl_2_O_4_, FeAl_2_O_4_, and CoAl_2_O_4_. Notably, the NiAl_2_O_4_ did not exhibit any active Raman modes within the analyzed wavenumber range. Both the polycrystalline and single-crystalline HE samples exhibit a blend of active Raman modes from their parent compounds, including E_g_, F_2g_, and A_1g_ modes. However, there was a notable distinction in the Raman spectra of the HEO samples—they show significant broadening and shifting of these modes compared to their parent compounds, as illustrated in Figure 6f. In the high-entropy samples, an F_2g_ mode is observed around 200 cm^–1^, reminiscent of that found in CoAl_2_O_4_. However, there are noticeable shifts in the E_g_ and F_2g_ modes, located approximately at 380 and 575 cm^–1^, respectively. The broadened and shifted Raman modes serve as direct evidence indicating significant lattice distortions within the high-entropy spinels. This observation aligns well with the anticipated behavior for HE compounds: the haphazard arrangement of A and B site atoms with varying masses and ionic radii throughout these compounds, reinforcing the notion of pronounced lattice distortions in these materials.

To probe deeper into the lattice distortion of (MgMnFeCoNi)Al_2_O_4_, they conducted Extended X-ray Absorption Fine Structure (EXAFS) measurements on this HEM and its corresponding parent compounds. Comparing the FT EXAFS data of the HE spinel phase with those of the parent compounds can unveil the extent of localized disorder present in the HE system. Figure 6g (i) illustrates the phase uncorrected FT spectra for the Co, Fe, and Mn K-edges within (MgMnFeCoNi)Al_2_O_4_. Notably, the peaks corresponding to metal–oxygen interactions between 1 and 2 Å display evident shifts in peak maxima. These shifts signify the distinct bond lengths associated with each measured absorber species within the system. Figure 6g (ii–iii) present a comparative analysis of the individual absorbers Mn and Co within the HE sample in contrast to their counterparts in the parent compounds MnAl_2_O_4_ (with partial inversion) and CoAl_2_O_4_ (exhibiting normal spinel structure). However, they endeavored to gather foundational insights into localized disorder by employing a theoretical model fitted to the ternary standard MnAl_2_O_4_, as depicted in Figure 6g (ii). Despite achieving a fit that closely aligns with the data for the metal–metal pairs, the outcomes are rendered invalid from a physical standpoint, as evidenced by the emergence of negative atom quantities. Based on these findings, it appears that there is an approximate equal distribution of Mn bonding sites in this standard, indicating partial inversion. This suggests a high level of disorder within the overall system, while showing approximately double the thermal expansion of its CoAl_2_O_4_ parent compound and significantly reduced thermal conductivity. Such discoveries enhance our understanding of how thermal expansion and transport are influenced by lattice distortions in HEMs.

We believe that to harness the benefits of HEOs, or the broader HEMs, to their fullest extent, it is essential to achieve thorough and uniform mixing of elements at the atomic level. This precise level of mixing should serve as the standard against which synthetic methods are evaluated. Understanding the statistical significance of this necessity entails recognizing the multitude of unique atomic sites present within a HEMs. Imperfect mixing leads to two significant drawbacks: First, the system fails to achieve maximal disorder, thus missing out on the complete advantages of entropic stabilization. Second, the exponential reduction in the number of theoretically unique sites hampers performance, especially in applications such as catalysis. Consequently, verifying whether various elements can combine to form a SS, as well as determining the potential crystal structures, can be an exceedingly time-consuming process. By utilizing calculation methods such as ab initio calculations and thermodynamic database approaches, it could be possible to improve efficiency by analyzing the atomic-scale crystal structures and physicochemical properties of synthetic materials.

## REVOLUTIONIZING COMPUTATIONAL APPROACHES: A NEW PERSPECTIVE ON METHODOLOGIES

Incorporating data-driven methodologies into the experimental phase of materials synthesis and optimization represents a feasible and logical advancement of materials informatics. The advent of HEOs, in line with HEAs, marked a radical departure from conventional theories and initiated new avenues for in-depth exploration in the field of material design. While computational studies have offered profound understandings into the formation, structures, properties, reactivity, and stability of simpler NP systems, addressing the compositional and structural complexities of HE systems remains stimulating, albeit increasingly feasible. The numbers of elements that might contribute to achieving a specific property goal can expand appreciably with the aid of computational tools. By employing high-throughput computation to analyze the enthalpies of formation of binary compounds, some models anticipate combinations of elements that are highly probable to generate single-phase HEOs.

Density Functional Theory (DFT) stands as a prevalent computational method utilized for exploring the theoretical aspects of new material system’s formation and characterizing the properties of synthesized materials. Some researchers utilized DFT to compute the adsorption energies on a random subset of the available binding sites located on the surface of the HEO. Then, using a simple machine learning algorithm, they forecasted the remaining adsorption energies and observed a strong agreement between the calculated and predicted values. Leveraging a comprehensive catalogue of available adsorption energies, they employed a suitable expression to predict catalytic activity and optimize the composition of HEOs. Again, Ting and co-workers investigated the relationship between concentration and structure in (MgZnMnCoNi)O_*x*_ and (CrFeMnCoNi)O_*x*_ HEO systems.^119^ They hypothesized that the metal oxides would form a SS based on solubility rules. Using DFT calculations, they determined the formation energy of the HEO, representing the energy difference between the HEO structure and its constituent elements in their ground states. Their findings showed that reducing the concentration of Mn (in Mn-poor HEO systems) favored the formation of an RS phase structure, whereas higher Mn concentrations (in Zn- or Ni-poor HEO systems) led to a spinel-phase structure. While Page et al. investigated the influence of local distortion in two pyrochlore-type HEOs, Nd_2_(Ta_0.2_Sc_0.2_Sn_0.2_Hf_0.2_Zr_0.2_)_2_O_7_ and Nd_2_(Ti_0.2_Nb_0.2_Sn_0.2_Hf_0.2_Zr_0.2_)_2_O_7+*x*_, employing a combination of simulation techniques alongside DFT-based ab initio molecular dynamics.^120^ These techniques included pair distribution function (PDF) analysis, reverse Monte Carlo (RMC) simulations, special quasi-random structures (SQS) generation, and Metropolis Monte Carlo simulations (MCS). Their findings revealed an average simulated crystal structure of orthorhombic (*Imma*), with the composition of cation species governing local distortions. For instance, Nd_2_(Ta_0.2_Sc_0.2_Sn_0.2_Hf_0.2_Zr_0.2_)_2_O_7_ exhibited a uniformly randomized structure, while Nd_2_(Ti_0.2_Nb_0.2_Sn_0.2_Hf_0.2_Zr_0.2_)_2_O_7+*x*_ displayed strong TiO_6_ octahedral distortion with a slight tendency toward short-range Ti clustering. These results underscored the significance of comprehending local distortion phenomena, offering insights into tailoring HEO properties for desired applications.

The demand for HEOs is not just driven by their scale and urgency, but also by the imperative to develop, execute, and implement them at a pace far quicker than the traditional multidecade timeline between discovery and commercialization. Thus, utilizing data-driven tools proves to be an efficient and effective approach for reliably managing HEO synthesis processes and gaining insights into their advantageous structure–property connections.

## APPLICATIONS

### Electrocatalysis

Electrocatalysis has become increasingly crucial in driving sustainable and environmentally friendly energy transformations.^121^ Metal oxides are among the most thoroughly studied electrocatalysts for their prospective technological applications in various energy generation processes. However, they have several limitations such as chemical and structural stability, poor electroconductivity, lower intrinsic activity, surface poisoning, etc. The presence of numerous elements in HEOs results in a vast array of atomic arrangements and surface microstructures with active catalytic elements. This diversity induces varying modes of adsorption for the reactants and associated intermediates. When elements are present in an atomically mixed form, even the electronic structures of the individual elements are likely to be modified. Thus, the HE of HEOs disrupts the immiscibility gap of elements, allowing robust control of elemental concentrations and promoting the optimization of catalytic properties. The surfaces offered by HEOs have a vast number of distinct binding site settings, resulting in a nearly continuous distribution of the corresponding adsorption energies. In this distribution, catalytic activity is predominantly influenced by sites possessing optimal properties, akin to how specific steps and microstrain on surfaces serve as crucial catalyst sites. Researchers have undertaken comprehensive studies, employing both experimental and theoretical approaches, to utilize HEOs for achieving effective and regulated electrochemical conversions of various small molecules, including H_2_O, O_2_, CO_2_, alcohols, and more. By maneuvering the composition of the HEOs to maximize the presence of sites featuring optimal adsorption energies, it is possible to enhance the catalytic activity more significantly. In this section of the Focus Review, we will briefly explore the utilization of HEOs across the domains of electrocatalysis for OER, hydrogenation of CO_2_, and oxidation of benzyl alcohol.

Exploring the electronic structure of HEOs is still in its preliminary stages, characterized by a nascent understanding. HEOs exhibit more isolated metal sites compared to alloys and display a broader array of structures, contributing to the complexity of this evolving field of research. For instance, in the case of (CeGdLaNdPrSmY)O_2−δ_, the observed band gap of 2.11 eV is notably smaller than the band gap exhibited by the CeO_2_ (3 eV) end member. While both Ce and Pr can assume a 4+ oxidation state, leading to the formation of substantial oxygen vacancies, the reduction in the band gap by 1 eV surpasses what would be anticipated solely from the presence of oxygen vacancies. It is hypothesized that the essential factor enabling this large band gap reduction is the Pr^4+^ oxidation state. This aligns with the observation that the ternary alloy (CeLaPr)O_2−δ_ also demonstrates a narrowing of the band gap. These investigations provide valuable insights into how a single element can exert a disproportionately influential impact on the electronic properties of a HEMs. But, to effectively manipulate the band gap of HEOs, a more thorough comprehension through both theoretical insights and experimental investigations is imperative.

**Figure 7:**
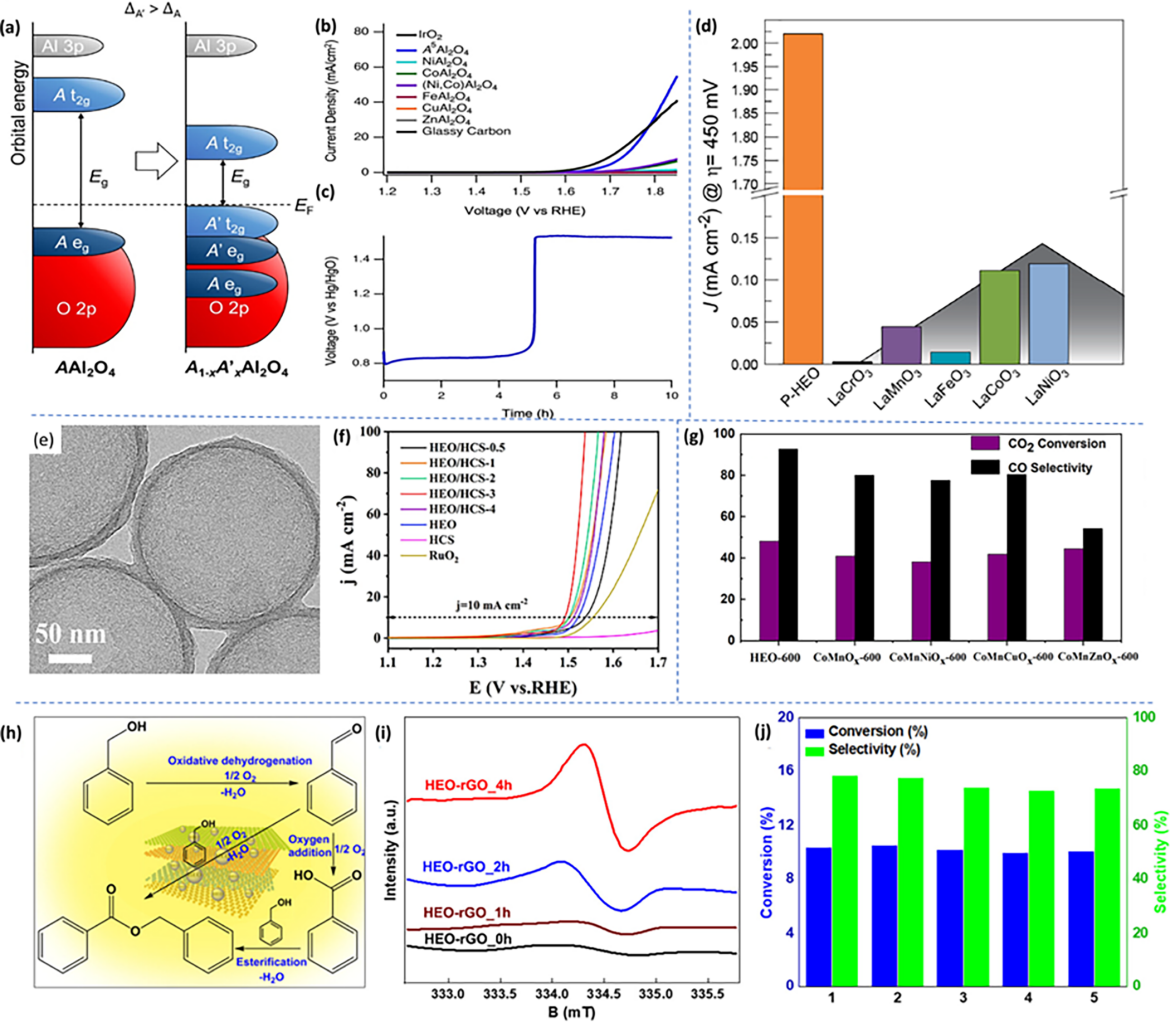
(a) Influence of TMs with differing electronegativities on the crystal field splitting (Δ) for tetrahedrally coordinated divalent Co and Cu. (b) Linear sweep voltammetry (LSV) data illustrating the OER in 1.0 M KOH for various samples: the AAl_2_O_4_ end members, (Co,Ni)Al_2_O_4_ SS, high-entropy A^5^Al_2_O_4_ spinel, and an IrO_2_ benchmark catalyst. (c) Chronopotentiometry data presenting the performance of the high-entropy A^5^Al_2_O_4_ spinel under a constant current density of 10 mAcm^–2^. Panels (a–c) are adapted with permission from ref (68). Copyright 2023 American Chemical Society. (d) Comparing the specific OER activities of P-HEO and its parent compounds involves examining the current density at a specific overpotential, in this case, 450 mV (equivalent to 1.68 V vs RHE). Panel (d) is adapted with permission from ref (64). Copyright 2023 American Chemical Society. (e) SEM image, and (f) OER performance of (FeCoNiCrMn)_3_O_4_ HEOs with hollow carbon spheres. Panels (e–f) are adapted with permission from ref (122). Copyright 2024 Elsevier. (g) Evaluation of the CO_2_ hydrogenation stability of HEO-600 and CoMnO_*x*_-600. Panel (g) is adapted with permission from ref (71). Copyright 2021 American Chemical Society. (h) Pictorial representation of the reaction mechanism for benzyl alcohol (PhCH_2_OH) oxidation using HEO-rGO catalyst. (i) EPR spectra of the oxidation reaction components at ambient temperature. (j) Recyclability test of the HEO-rGO catalyst for the solvent-free oxidation of PhCH_2_OH. Panels (h–j) are adapted with permission from ref (123). Copyright 2024 American Chemical Society.

Schaak and co-workers designed an A^5^Al_2_O_4_ type spinel, (Fe_0.2_Co_0.2_Ni_0.2_Cu_0.2_Zn_0.2_)Al_2_O_4_, and its end members by a solution combustion method and evaluated their band gaps using diffuse reflectance measurements.^68^ They found that the band gap narrowed to 0.9 eV for the A^5^Al_2_O_4_ spinel, much lower than those of all other end members. The emergence of this phenomenon was attributed to variations in electronegativities (χ) and Δ between the dopant and host transition-metal cations. These distinctions lead to the introduction of 3d states within the band gap of the host. Moreover, TM dopants with lower Δ can influence the *E*_g_ by diminishing the crystal field splitting of the host TM. In practical terms, this lowers the energy of unoccupied t_2g_ states, subsequently reducing the band gap energy, as depicted in Figure 7a. The impact of this effect becomes more pronounced with an increasing difference in the Δ between the host and dopant TMs. Notably, enhancing the proportion of the TM with lower Δ amplifies this effect. Considering that the synergistic effects arising from the mixed metal surface create intricate active sites for the OER, and the band gap narrowing, they performed the LSV of HE A^5^Al_2_O_4_ spinel in 1.0 M KOH and compared it with the corresponding end members, as presented in Figure 7b. At an applied potential of 1.7 V vs the RHE, A^5^Al_2_O_4_ surpassed all individual metal end members by achieving a current density of 10 mA cm^–2^ at an overpotential of 400 mV. The A^5^Al_2_O_4_ spinel exhibited competitive activity with IrO_2_, a costly precious metal compound widely recognized as a benchmark catalyst for the OER.

Additionally, the stability of the HE A^5^Al_2_O_4_ spinel was assessed through chronopotentiometry, a technique where the electrode is maintained at a constant current, and potential changes are monitored over time. A consistent potential indicates stable catalytic performance. In Figure 7c, the data reveals that the A^5^Al_2_O_4_ spinel maintained a constant potential for around 5 h when subjected to a current density of 10 mAcm^–2^. However, it then experienced a sudden increase in voltage, signaling instability. This instability may stem from potential leaching, which aids the formation of a passivating layer that blocks the HEO surface. Hence, the increased conductivity resulting from the narrowed band gap is beneficial for catalytic processes.

Kante et al. synthesized the perovskite HEO LaCr_0.2_Mn_0.2_Fe_0.2_Co_0.2_Ni_0.2_O_3-δ_ (P-HEO) and investigated the role of multication composition in enhancing catalytic activity for the OER.^64^ This reaction is crucial in various electrochemical energy conversion technologies, such as green hydrogen generation. When comparing the activity of the (001) facet of LaCr_0.2_Mn_0.2_Fe_0.2_Co_0.2_Ni_0.2_O_3−δ_ with its parent oxides (single B-site in the ABO_3_ perovskite), distinct trends emerge. While the single B-site perovskites exhibit activity that roughly adheres to expected volcano-type patterns predicted from DFT, the designed HEO significantly surpasses all its parent compounds, as shown in Figure 7d. The HEO demonstrated a remarkable enhancement, ranging from 17 to 680 times higher currents at a fixed overpotential, highlighting its superior performance. Since all samples were grown as epitaxial layers, these findings revealed an inherent relationship between composition and function, circumventing the influence of complex geometries or uncertain surface compositions. The remarkably elevated OER activity underscores the appeal of HEOs as a promising material class, abundant in Earth’s resources, for highly active OER electrocatalysts. This potential suggests the feasibility of fine-tuning activity beyond the scaling limits observed in mono- or bimetallic oxides.

Wang and co-workers achieved successful synthesis of a composite catalyst of HEO through a facile method involving microwave-assisted solvothermal synthesis coupled with annealing.^122^ Their approach combined (FeCoNiCrMn)_3_O_4_ NPs with hollow carbon spheres (illustrated in Figure 7e). This innovative design led to the uniform dispersion of NPs, boasting an average size of approximately 3.3 nm. The resulting nanocomposite, characterized by its substantial surface area, facilitated efficient mass transfer and gas release while maximizing exposure of active surface sites. This enhancement significantly boosted the electrocatalytic activity toward OER (Figure 7f). Again, Zhao et al. reported that, in their study, the in situ formation of CuCoNi nanoalloys on a Co_3_MnNiCuZnO_*x*_ HEO matrix has been utilized to create a sintering-resistant interface between metal and oxide phases for the CO_2_ hydrogenation reaction.^71^ The HE catalyst, consisting of a single reverse spinel structure of Co_3_MnNiCuZnO_*x*_, was synthesized via a mechanochemical redox-based approach followed by thermal treatment at 600 °C. The significant presence of oxygen vacancies and exposed active sites, including CuCoNi nanoalloys and metal-oxide interfaces, in a reducing atmosphere facilitated hydrogen dissociation and CO_2_ activation. This led to the demonstration of high catalytic activity, with HEO-600 achieving 48% CO_2_ conversion and 94% CO selectivity in the RWGS reaction at 500 °C, as demonstrated in Figure 7g. Additionally, the highly dispersed active NPs on the multimetal oxide matrix played a crucial role in enhancing the catalytic performance during the RWGS process, ensuring excellent stability over a period of 100 h for Co_3_MnNiCuZnO_*x*_.

However, Mehrabi-Kalajahi et al.’s study outlined the synthesis of noble metal-free NPs composed of (CoFeMnCu-NiCr)_3_O_4_ HEO and their subsequent grafting onto reduced graphene oxide (rGO) to form a HEO-rGO composite material.^123^ The HEO-rGO nanocomposites exhibited exceptional performance in the aerobic and solvent-free oxidation of PhCH_2_OH. The most probable pathway for the oxidation of PhCH_2_OH involves the generation of free radicals during the reactions (illustrated in Figure 7h). Through careful adjustment of catalytic reaction conditions such as temperature, pressure, and time, the selectivity toward benzaldehyde as the primary product was optimized. The catalyst’s synthesis resulted in abundant surface oxygen vacancies and exposed catalytic active sites, which significantly boosted the conversion rate to 10.36% with a selectivity of 78.5% toward benzaldehyde at 100 °C within just 4 h. EPR measurements were employed to showcase the radical-based mechanism of the oxidation reaction, with a focus on monitoring the organic free radical. As depicted in Figure 7i, no EPR signal is initially observed at the onset of the catalyst-assisted oxidation of PhCH_2_OH in the absence of solvent. But, over prolonged reaction times, an EPR signal emerges, with its intensity escalating as the reaction progresses. Figure 7j illustrates the assessment of the stability and reusability of the HEO-rGO catalyst during the aerobic oxidation of PhCH_2_OH. Across five cycles of the oxidation reaction under 10 atm air pressure at 100 °C for 4 h, the conversion rate of benzyl alcohol experienced a slight reduction from 10.36% to 10.12%. Similarly, the selectivity toward benzaldehyde decreased from 78.5% to 73.8%. This modest decrease in conversion and selectivity rates is attributed to a minor loss in active surface area of the composite catalyst, possibly due to the agglomeration of HEO catalysts on the surface of rGO.

Thus, HEOs demonstrates superior performance in catalyzing diverse electrochemical reactions. This enhanced catalytic capability can be attributed to the broader range of compositional adjustments available with HEOs, surpassing the limitations of miscibility observed in conventional oxide nanomaterials.

### HEOs in Lithium-Ion Battery (LIB)

LIBs have emerged as the leading power source for electric vehicles and green grid energy storage. The conventional metal oxides and transition metal oxides (TMOs) are good alternatives for LIBs because they can serve as both electrode materials and fast-ion conductor electrolytes. Despite their high energy density, safety concerns have significantly challenged their widespread adoption. This propelled researchers to look for alternative nanomaterials that could replace the current electrodes and electrolytes used in LIBs. In this part, we will offer a concise outline of the application of HEOs in LIBs.

#### HEOs as Li-Ion Anode Battery

HEOs offer significant value in battery applications due to their ability to be finely tuned for short-range order, energy landscape optimization, volumetric stability, and chemical versatility. These enhancements make them exceptionally beneficial for improving battery performance and durability, especially for rechargeable batteries. They have garnered growing interest, particularly for their potential as LIB anodes. These materials offer several advantages beyond the inherent multielectron redox mechanism and safety features of TMOs: (i) Versatile component design reduces the reliance on any single element and offers new avenues for customizing electrochemical behavior. (ii) The cock-tail effect and kinetic diffusion characteristics of HEOs enable them to sustain structural integrity when subjected to harsh operating conditions. Such stability under stress minimizes electrode volume expansion and enhances cycling durability. (iii) HEOs possess a complex surface with multielement synergy, providing continuous adsorption energy ideal for multistep tandem reactions. (iv) The presence of abundant internal defects within the highly disordered and distorted lattice of HEOs facilitates the essential migration of electrons and ions, vital for optimizing energy storage efficiency. (v) By modifying the stoichiometry, the electronic structure of HEOs, including the Fermi level relevant to the electrode potential, can be adjusted, offering tunability for enhanced performance.

Sarkar et al. pioneered the utilization of (MgCoNiCuZn)O as anodes in LIBs and investigated its lithium storage mechanism using in situ XRD and SAED.^124^ During the initial discharge, the characteristic diffraction peaks of the RS structure gradually diminished. However, the reflection of the RS phase persisted in SAED, indicating a transformation from large particles (poly-/nanocrystallites) of (MgCoNiCuZn)O into smaller crystallites over time. The early breakthroughs in maximizing the advantages of extensive composition flexibility through the fine-tuning of cation stoichiometry and species incorporation in HEOs motivated other researchers to investigate the mechanistic insights behind designing these electrode materials with both high capacity and durability.

**Figure 8:**
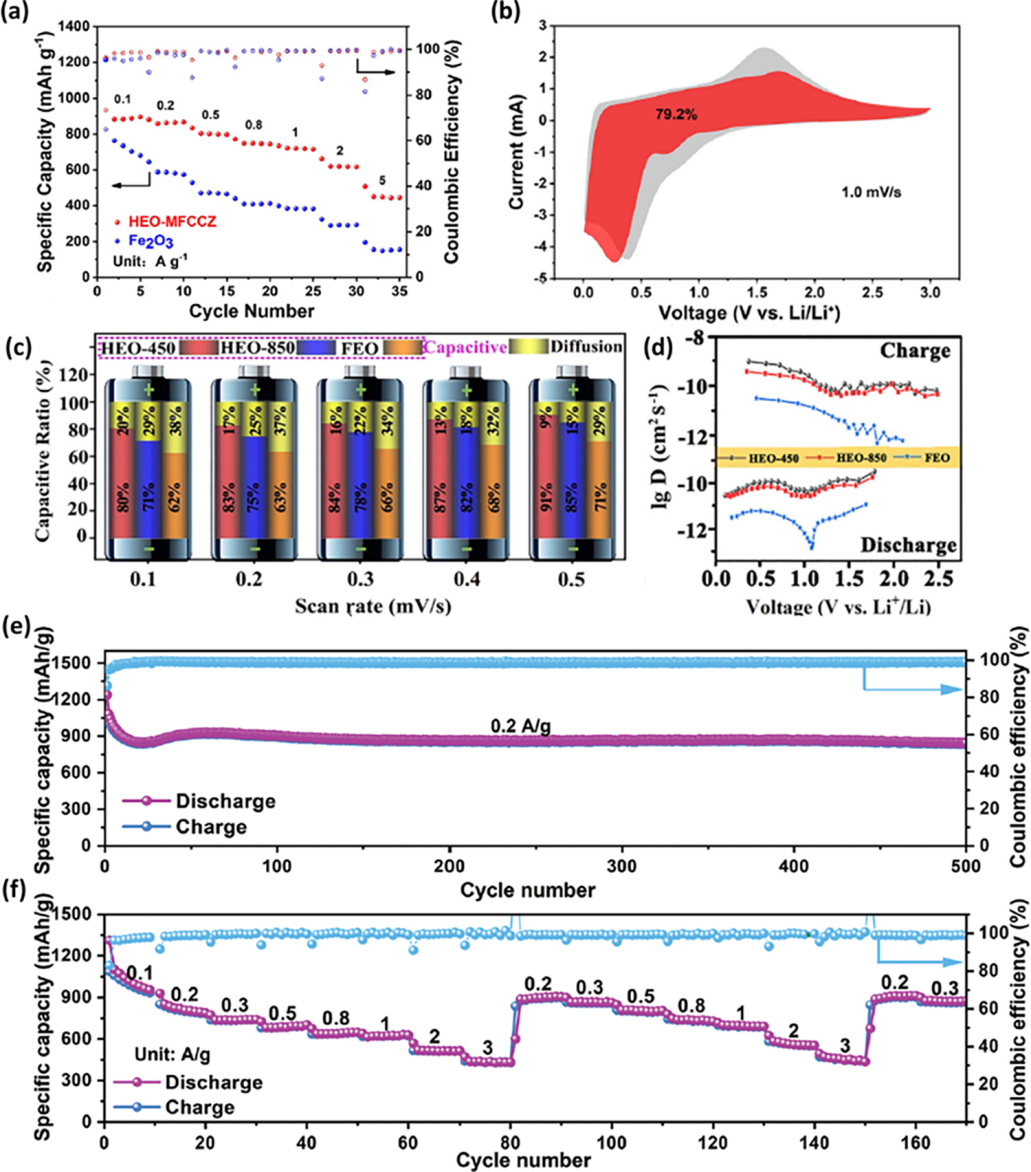
(a) Comparison of the rate performance of the (Mn_0.23_Fe_0.23_Co_0.22_Cr_0.19_Zn_0.13_)_3_O_4_ and its parent material, Fe_2_O_3_. (b) Pseudocapacitance contribution percentage of the HEO electrode at a scan rate of 1.0 mV s^–1^. Panels (a–b) are adapted with permission from ref (83). Copyright 2023 Royal Society of Chemistry. (c) The relative contribution of diffusion-controlled and capacitive-controlled capacities vary across different scan rates and (d) lithium-ion diffusion coefficients during the charge/discharge process for electrodes HEO-450, HEO-850, and FEO. Panels (c–d) are adapted with permission from ref (125). Copyright 2022 American Chemical Society. (e) Galvanostatic rate capabilities and Coulombic efficiency of M_3_O_4_-based half-cells. (f) Ultralong cycling performance and Coulombic efficiency of M_3_O_4_-based half-cells with 2000 cycles at 3 A g^–1^. Panels (e–f) are adapted with permission from ref (126). Copyright 2023 John Wiley and Sons.

Leng et al. reported a phase pure spinel HEO of (Mn_0.23_Fe_0.23_Co_0.22_Cr_0.19_Zn_0.13_)_3_O_4_ and assembled it into coin cells to explore their lithium storage performance.^83^ During the anode cycling process, when this material transformed into a coexisting state of amorphous and nanocrystalline structures, the electrochemically inactive Zn triggered a pegging phenomenon, resulting in heightened stability, while also introducing defect sites. The rate performance of the as-obtained materials and its parent, Fe_2_O_3_, is depicted in Figure 8a. In comparison to Fe_2_O_3_, the HEO material exhibited superior discharge specific capacities at various current densities: 890, 864, 798, 746, 722, 619, and 448 mA h g^–1^ at 0.1, 0.2, 0.5, 0.8, 1, 2, and 5 A g^–1^, respectively. Also, the HEO electrode showcased remarkable resilience to fluctuating rates and exhibits reduced polarization. Consequently, it displayed subtle alterations in the shape of charge/discharge curves across varying current densities, both low and high. Figure 8b illustrates the percentage of pseudocapacitive contribution for both materials when subjected to a scan rate of 1 mV s^–1^. The data revealed that the HEO electrode exhibited a notably high pseudocapacitive contribution percentage, reaching approximately 79.2%. This observation provides significant insights into the mechanisms underlying lithium storage and lays a solid foundation for the development of novel anode materials tailored for advanced LIBs.

Yang et al. employed a sol–gel method for the low temperature synthesis of porous HEO (Cr_0.2_Fe_0.2_Co_0.2_Ni_0.2_Zn_0.2_)_3_O_4_ in spinel-phase.^125^ The distinctive porous HEO nanostructure served a dual purpose: facilitating the movement of the electrolyte while also mitigating the volume fluctuations of active materials during cycling. Figure 8c illustrates the ratio of capacitance contributions from HEO-450, HEO-850, and FEO electrodes to the overall contributions within the scan rate range of 0.1 to 0.5 mV/s. Notably, it is observed that the capacitance contribution of the HEO-450 electrode surpasses those of HEO-850 and FEO electrodes across all scan rates. This observation underscores the enhanced capacitance contribution of the HEO-450 electrode, signifying its pivotal role in enhancing rate performance. Throughout the entire charge/discharge cycle, the HEO-450 electrode exhibited the highest diffusion coefficient. This enhanced diffusion coefficient in HEO electrodes is attributed to the greater disorder within the HEO material itself, leading to higher diffusion coefficients of lithium-ion (Figure 8d) values. Additionally, the stabilizing influence of entropy hampers the formation of cation short-range order within the crystalline structure of HEO through lattice distortion. This effect ensures rapid lithium-ion transport, leading to outstanding electrochemical performance.

Again, Hou and colleagues successfully synthesized a spinel HEO, with the composition (Co_0.2_Mn_0.2_V_0.2_Fe_0.2_Zn_0.2_)_3_O_4_.^126^ Remarkably, when employed as an anode material, the spinel HEO exhibited exceptional reversible cycling durability, which was primarily attributed to its ability to recover its initial spinel phase over multiple cycling events. In situ TEM analysis revealed that following the initial lithiation process, the nanomaterials experienced a notable increase in both projected area (∼41%) and volume (∼68%). Such controlled volume expansion is crucial, as it maintains the high specific capacity of the spinel HEO anode while preserving its mechanical integrity, ensuring stable performance. Thus, the convergence of factors such as elevated configurational entropy, presence of high-valent metal cations, and the structural attributes of the spinel configuration is identified as the underlying mechanism driving the development of a robust and high-capacity anode with enduring stability (Figure 8e–f). Moreover, prior investigations have demonstrated that magnetic metal atoms like Fe, Co, and Ni possess the capability to store a significant number of spin-polarized electrons. As a result, these metal atoms have the potential to augment the overall capacity during discharge at lower potentials.

#### HEOs as Li-Ion Cathode Battery

**Figure 9:**
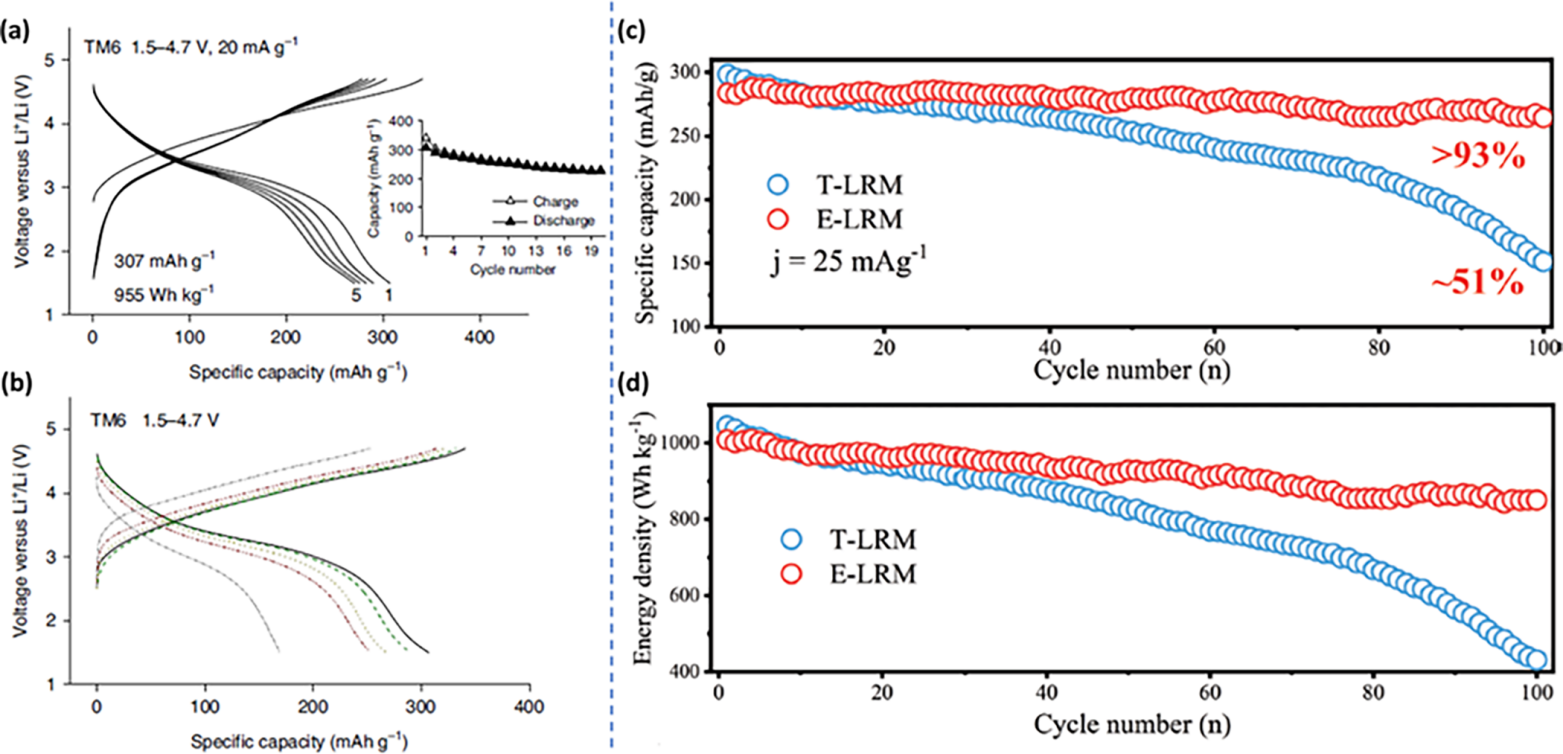
(a) Electrochemical Voltage profile of TM6 (1.5–4.7 V at 20 mA g^–1^). (b) Rate capability of TM6 cycled between 1.5 and 4.7 V. Panels (a–b) are adapted with permission from ref (127). Copyright 2021 Springer Nature. (c) Electrochemical capacity retention comparison and (d) energy retention of T-LRM and E-LRM after 100 cycles at 0.1 C. Panels (c–d) are adapted with permission from ref (. Copyright 2023 John Wiley and Sons.

The change in entropy of electrodes plays a crucial role in influencing the heat generation within batteries, thereby exerting a substantial impact on their overall performance. Through a comparative analysis of cation-disordered RS (DRX)-type cathodes containing 2, 4, 6, and 12 TM species, Ceder and colleagues revealed a consistent trend: as the number of TM cation species increases, short-range order decreases, while energy density and rate capability consistently improve.^127^ To ensure optimal Li-ion transmission performance, each material was formulated with an excess of 30 wt % Li, while 15% of the O content was substituted with fluorine. But, as the diversity of elements expanded, the HE effects intensified, increasing the degree of disorder among elements. Consequently, this mitigated the influence of the short-range ordered structure on Li-ion transmission. Figure 9a presents the voltage profile of six TM species (TM6) within the voltage window of 1.5–4.7 V at 20 mA g^–1^ (inset: corresponding capacity retention plots). Although the charge profiles bear similarities to those of Mn^3+^/Mn^4+^-redox-based counterparts, the discharge profiles exhibit a more gradual slope. Figure 9b demonstrates the rate capability of TM6 through its first-cycle voltage profiles while being cycled between 1.5 and 4.7 V at various current densities: 20, 50, 200, 500, and 2,000 mA g^–1^. Based on this, it is evident that a DRX cathode incorporating TM6 achieved a capacity of 307 mAh g^–1^ (equivalent to 955 Wh kg^–1^) at a low rate of 20 mA g^–1^. Furthermore, it maintains a capacity of over 170 mAh g^–1^ during cycling at a high rate of 2,000 mA g^–1^. It must be noted that the number of elements rather than the specific combination of cations, significantly influenced the capacity and Li-ion transmission of the cathode. However, once the variety of elements surpassed a threshold, further increase had minimal effect on cathode performance. This could be attributed to the complete suppression of local short-range order. Again, based on their previous work^19,20,128^ and others,^129^ Song et al. observed that, with an increase in the intralayered configuration entropy, better cycle stability can be attained. Thus, they designed entropy-stabilization-strategy enhanced Li-rich cathode material (E-LRM) Li_1.0_(Li_0.15_Mn_0.50_Ni_0.15_Co_0.10_Fe_0.025_Cu_0.025_Al_0.025_Mg_0.025_)O_2_.^130^ The initially prepared E-LRM achieved an impressive energy density exceeding 1000 Wh kg^–1^, accompanied by an initial Coulombic efficiency of approximately 85%. This efficiency surpasses that of a typical Li-rich cathode, Li_1.20_Mn_0.54_Ni_0.13_Co_0.13_O_2_ (T-LRM), which stands at 80%. The material demonstrated a remarkable capacity of over 260 mAh g^–1^, retaining more than 93% of its capacity even after 100 cycles at a current density of 0.1 C. In contrast, the T-LRM experiences rapid capacity degradation, with only about 150 mAh g^–1^ capacity remaining and a retention rate of 51% (Figure 9c). Additionally, its energy density retention is merely 40%, significantly lower than that of the E-LRM, which retains more than double the energy density (Figure 9d). Hence, introducing multiple elements, the local structural diversity and distortion energy of Mn^4+^ are heightened, as confirmed by the findings of DFT calculations. This augmentation results in enhanced local structural adaptability and improved stability.

#### HEO Based Solid-State Electrolytes (SSEs) of Lithium Battery

The burgeoning interest in solid-state batteries has spurred extensive research into oxide ceramic electrolytes. Nevertheless, improving the ionic conductivity of these ceramic electrolytes at room temperature continues to pose a significant challenge. The utilization of HEOs as electrolytes offers a promising avenue for addressing these challenges. Specifically, the introduction of the HE effects notably improves the stability of the electrolytes. Consider garnet-type electrolytes, known for their high ionic conductivity (σ) and wide electrochemical window, yet vulnerable to instability in ambient air. By harnessing the high-entropy effect, the phase stability of SSEs can be significantly enhanced. Han and colleagues achieved this by successfully synthesizing an HE garnet, Li_6.2_La_3_(Zr_0.2_Hf_0.2_Ti_0.2_Nb_0.2_Ta_0.2_)_2_O_12_, using the solid-state reaction method.^131^ The HE effects significantly enhance phase stability, thereby greatly improving the electrolyte’s resilience in ambient air. Unlike traditional LLTO (Li_3*x*_La_2/3–*x*_TiO_3_), which tends to produce considerable LiCO_3_ when exposed to air, the HE garnet exhibits remarkable stability. Again, the study on HEOs opened an innovative platform for enhancing the conductivity of SSEs. For instance, Palakkathodi Kammampata et al. enriched the garnet LLZO structure by incorporating various alkaline earth metal elements, resulting in the fabrication of a series of single-phase materials like Li_6.5_La_2.9_A_0.1_Zr_1.4_Ta_0.6_O_12_ (where A = Ca, Sr, Ba).^132^ Such HEMs exhibited exceptional room temperature ionic conductivity, exceeding that of previously reported conductive materials by 1 order of magnitude.

While HEOs show promise, not all of them necessarily enhance ionic conductivity. Bonnet and colleagues addressed this by modulating the concentration of vacancies in the nanomaterial through adjustments in the added ion radius, aiming to enhance ionic conductivity.^133^ Yet, even with these efforts, the ionic conductivity of (Hf_1/3_Ce_1/3_Zr_1/3_)_1–*x*_(Gd_1/2_Y_1/2_)_*x*_O_2–*x*/2_ remained below 4 × 10^–4^ S cm^–1^ at 600 °C. Consequently, advancing solid HEO electrolytes requires a combination of theoretical backing and extensive experimental trials to refine existing solid electrolyte materials.

## FUTURE AVENUES FOR EXPLORATION OF HEOs

Given their inherent complexity, there is a need for contemplation regarding the prospective research endeavors that will be essential in the future. Therefore, the prospects underpinning advanced understanding of HEOs, navigating the synthesis for targeted surface compositions and atomic arrangements, have been outlined to propel further progress in the field.

The computational aspect of HEOs typically lags the experimental progress. Relying on chemical intuition is usually effective in predicting the formation of a single phase from constituent oxides. Often, the empirical approach of mixing oxides in the laboratory through trial and error is still faster than relying solely on *in silico* predictions. While generic methods can be devised to assess the feasibility of forming HEMs in a single phase, using first-principle calculations, we believe that the computational objective is not only to identify what can be synthesized but also to determine what should be synthesized. Also, methods for modeling disordered systems are not as advanced as those for ordered structures.

There are vast opportunities beyond the conventional equimolar composition, where maximum configurational entropy (beyond the current limit) can be achieved. The design of experiments (DoE) can serve as a chemometric tool, aiding in the strategic selection of a restricted set of experiments. The responses from these experiments are subsequently leveraged to comprehensively map the entire experimental domain. This process is instrumental in the exploration of novel materials, aiming to unlock distinctive properties and potential applications. The DoE outcomes in the recent past have revealed that the stability range of the RS HEO can extend well beyond the equimolar composition, Mg_0.20_Ni_0.20_Co_0.20_Cu_0.20_Zn_0.20_O.^82^ Larger fractions of Cu^2+^ and Zn^2+^ can be successfully incorporated into the RS structure, maintaining a single phase. This occurs even in the case of a lower configurational entropy and a more substantial enthalpic contribution. These discoveries not only raise questions about the influence of configurational entropy on the formation of the RS phase HEOs but also underscore the need to delve into a more comprehensive exploration to deepen our understanding of entropy’s role—in terms of both stability and functional properties. Furthermore, special emphasis must be laid on utilizing machine-learning tools, that facilitate the mapping of categorical synthetic outcomes such as crystal phase determination, to build predictive models, analyze large data sets, and integrate the diverse data sources with DFT calculations. Such integration offers a methodology for refining interatomic potentials by leveraging data sets generated through DFT techniques. This can significantly reduce the time and resources needed for experimental synthesis and property optimization.

The domain of HEO is younger than HEA, and numerous successful synthesis pathways, like the carbothermal shock method, solution combustion, etc., initially designed for achieving phase pure HEAs, have been adapted for the synthesis of HEOs. However, these techniques often necessitate the requirement of sophisticated equipment and lack scalability, thus impeding their commercialization. Furthermore, these methodologies impose constraints on the manipulation of composition and morphology, crucial factors in NP materials. This limitation is particularly essential in catalysis, where the shape of particles can exert influence on catalytic mechanisms, resulting products, and distribution of products.

Although achieving entropy stabilization in the synthesis of colloidal HE materials often poses challenges due to the requirement for elevated temperatures, we foresee vast opportunities on this horizon with a wide range of compositions and involving a large number of elements. By adopting a combinatorial synthesis approach, for instance, intensive chemical manipulation of these materials assisted by galvanic replacement methods, there is potential to advance the creation of intricate and target-specific HEOs in bulk scale. Also, designing inorganic complexes of various metal precursors, for instance single source metal precursors, could streamline and simplify materials synthesis pathways by enabling the simultaneous nucleation of metal precursors and afford phase pure growth at moderate temperatures. Not only will such synthetic innovation reduce production cost, by doing away with the expensive synthesis methods such as CTS methods, but it will also enable enhanced control over the structure and properties of HEOs. The micro-nano structured HEOs could introduce unique size effects and surface/interface phenomena. For instance, the shape, specific surface areas, and exposed surfaces of HEOs can be tailored through colloidal or sol–gel synthesis methods. Furthermore, there is considerable potential for further exploration concerning HEO quantum dot NCs, both in a broader context of assessing crystalline nanomaterials across the periodic table for their capacity to form HE phases through a precise atom-by-atom assembly method and in fine-tuning the morphology and particulate size to modulate the physical characteristics of these innovative systems. A significant and profound implication stemming from this research lies in the exciting prospect of introducing novel categories of nanomaterials, potentially exhibiting emergent properties arising from the combined effects of the HE cocktail effect and quantum confinement. Such synthetic breakthroughs will open possibilities for designing innovative nanodevices with improved performance characteristics tailored for practical applications in the real world.

In a large number of existing literature reports on HEOs (or inorganic HEMs), there seems to be a general lack of comprehensive insights into atomic-level mixing and the strategies for optimizing it to generate entropy-stabilized compounds. While the traditional characterization techniques like power XRD, SEM, TEM, and XPS can assist in identifying fundamental phase structures, morphologies, elemental distributions, and valence states, they may fall short in achieving the necessary resolution to untangle the complex mixing of multiple elements. For instance, utilizing synchrotron X-ray-based methods with significantly shorter wavelengths holds the potential to offer enhanced resolution for a more insightful comprehension of the atomic arrangement, bonding, coordination, and electronic properties of HEOs. Again, in most cases, the synthesized inorganic nanomaterials are characterized with STEM-EDX spectroscopic maps, which solely reveal the distribution of elements at the microscale, lacking clear evidence of homogeneous mixing at the atomic level. The most promising avenue for higher-throughput and higher-resolution needs may lie in four-dimensional STEM (4D-STEM) microscopy techniques. These methods involve capturing sequences of 2D diffraction patterns at various nanometer-scale positions, employing direct electron detectors that operate at a frame rate reaching thousands of frames per second for fast characterization of local lattice distortion, short-range ordering, defects, and structural heterogeneity. HEO electrocatalysts encounter several challenges. There remains a lack of understanding regarding configuration entropy and the intricate nature of reaction mechanisms. Moreover, these catalysts are susceptible to corrosion when exposed to acidic environments.

Unlike alloys, oxides exhibit a broader range of crystal structures and local site symmetries. Their inherent stability, coupled with interactions mediated through the oxygen sublattice, adds to their appeal for research. Additionally, the technological significance of oxides further emphasizes the value of exploring HEOs.

Dislocations, as line defects within the crystal lattice, play a pivotal role as the primary agents responsible for facilitating plastic deformation in crystalline metals. Many defects such as vacancies, grain boundaries, etc. hinder the plasticity of HEMs, thereby making the material stronger. Thus, the highest degree of strengthening occurs when robust obstacles are closely spaced. However, in contrast to conventional metals, where strengthening typically comes at the expense of ductility and toughness, certain HEMs exhibit a unique ability to avoid this trade-off. It seems that specific, yet unidentified, features of twin and phase boundaries in these HEMs contribute to maintaining resilience even with increased strength. A thorough understanding of the factors influencing this behavior is essential for strategically designing HEOs that possess both increased strength and enhanced toughness. This advancement promises to unlock a plethora of new materials with applications spanning various fields such as optoelectronics, (photo)electrocatalysis, photonics, and thermoelectric energy generation.

In addition to their role as robust and enduring materials in various energy storage and conversion systems, HEOs doped with alkali metal cations can be considered as potential SSEs for alkali ion and alkali sulfur batteries. This is attributed to their remarkable ionic conductivity properties, which make them promising candidates for facilitating efficient ion transport within battery systems.

## SUMMARY AND PROSPECTS

The discovery of HEMs has nucleated as an unusual area of inquiry at the forefront of inorganic nanomaterials, their advanced characterizations, and functionality. This current Focus Review provides a comprehensive idea of HEMs with special emphasis on HEOs and how the synthesis innovations of these seeds have rapidly grown to current research interest, which tested the limits of material synthesis, instrumentation, computation, and imagination. The continued research endeavors in this area have the potential to result in the commercialization of HEOs for a broad spectrum of structural and energy applications, offering improved performance and efficiency in contrast to conventional oxides and nanomaterials. In brief, the review offers a foundational insight into HEOs and explores the impact of recent discoveries made in the past three years on the functionalization and application of these materials in catalysis and batteries. Nevertheless, the extensive array of potential compositions and intricate atomic configurations poses severe challenges for synthesis, high-resolution characterization, comprehension, and practical application of HEOs. For instance, the numerous principles derived from HEAs can be directly employed in the context of oxides, but the complexity of local distortions in HEOs arises from the intricate interplay of the oxygen sublattice, along with the challenges associated with balancing charges between the anionic and cationic sublattice. This phenomenon is distinct from HEAs and needs detailed investigation. Again, the numerous possible arrangements of surface atoms make it challenging to identify the true active centers by investigating just one or a few models. In the development of HEOs, it is also essential to consider cost factors and sustainability. This involves assessing not only the costs associated but also the practicality of scrap recovery and recycling as part of the overall considerations. To enhance cost-effectiveness, it is imperative to embrace innovative strategies for producing HEOs from non-novel, earth abundant elements.

Likewise, being at an initial stage, these complex materials present several other formidable challenges. Thus, it is essential to emphasize that significant progress in resolving the structural stability principles of HEOs and understanding the identities and functions of surface species, as well as their transformations under operational conditions, can only be achieved through active collaboration between experimental and theoretical researchers. This integrated strategy promises to expedite the exploration and creation of top-tier materials, paving the way for transformative technological innovations.

Even though unravelling the synthesis–structure–property dynamics in this complex HEO is a formidable challenge, profound understanding of their physical, chemical, and mechanical properties in a multidimensional space is essential for steering material design and optimization further. HEOs are poised to play a leading role in the field of structural, and potentially functional, materials for at least another decade, if not longer. The interrelated domains in HEMs should persist in drawing inspiration from each other, working collectively toward the overarching objective of comprehending the genuine influence of entropy in the realm of HEOs.

However, a universal catalyst material capable of facilitating multiple reactions simultaneously does not yet exist. Nevertheless, HEOs present a promising approach toward achieving such universality. HEO catalysts consist of a surface with a diverse array of multimetal atoms, which remain thermally stable and active, potentially enabling the facilitation of various reactions concurrently.

## References

[ref1] George E. P.; Raabe D.; Ritchie R. O. High-entropy alloys. Nat. Rev. Mater. 2019, 4, 515–534. 10.1038/s41578-019-0121-4.

[ref2] Zhou M.; Li C.; Fang J. Noble-metal based random alloy and intermetallic nanocrystals: syntheses and applications. Chem. Rev. 2021, 121, 736–795. 10.1021/acs.chemrev.0c00436. 32902963

[ref3] Chen P.-C.; Liu X.; Hedrick J. L.; Xie Z.; Wang S.; Lin Q.-Y.; Hersam M. C.; Dravid V. P.; Mirkin C. A. Polyelemental nanoparticle libraries. Science 2016, 352, 1565–1569. 10.1126/science.aaf8402. 27339985

[ref4] Yao Y.; Dong Q.; Brozena A.; Luo J.; Miao J.; Chi M.; Wang C.; Kevrekidis I. G.; Ren Z. J.; Greeley J.; Wang G.; Anapolsky A.; Hu L. High-entropy nanoparticles: Synthesis-structure-property relationships and data-driven discovery. Science 2022, 376, eabn3103 10.1126/science.abn3103. 35389801

[ref5] Amiri A.; Shahbazian-Yassar R. Recent progress of high-entropy materials for energy storage and conversion. J. Mater. Chem. A 2021, 9, 782–823. 10.1039/D0TA09578H.

[ref6] Wu D.; Kusada K.; Yamamoto T.; Toriyama T.; Matsumura S.; Kawaguchi S.; Kubota Y.; Kitagawa H. Platinum-group-metal high-entropy-alloy nanoparticles. J. Am. Chem. Soc. 2020, 142, 13833–13838. 10.1021/jacs.0c04807. 32786816

[ref7] Miracle D. B.; Senkov O. N. A critical review of high entropy alloys and related concepts. Acta Mater. 2017, 122, 448–511. 10.1016/j.actamat.2016.08.081.

[ref8] Buckingham M. A.; Skelton J. M.; Lewis D. J. Synthetic strategies toward high entropy materials: atoms-to-lattices for maximum disorder. Cryst. Growth Des. 2023, 23, 6998–7009. 10.1021/acs.cgd.3c00712. PMC10557048 37808901

[ref9] Zhang L.; Jia J.; Yan J. Challenges and strategies for synthesizing high performance micro and nanoscale high entropy oxide materials. Small 2024, 2309586 10.1002/smll.202309586. 38348913

[ref10] Moniri S.; Yang Y.; Ding J.; Yuan Y.; Zhou J.; Yang L.; Zhu F.; Liao Y.; Yao Y.; Hu L.; Ercius P.; Miao J. Three-dimensional atomic structure and local chemical order of medium- and high-entropy nanoalloys. Nature 2023, 624, 564–569. 10.1038/s41586-023-06785-z. 38123807

[ref11] Löffler T.; Savan A.; Garzón-Manjón A.; Meischein M.; Scheu C.; Ludwig A.; Schuhmann W. Toward a paradigm shift in electrocatalysis using complex solid solution nanoparticles. ACS Energy Lett. 2019, 4, 1206–1214. 10.1021/acsenergylett.9b00531.

[ref12] Yeh J. W.; Chen S. K.; Lin S. J.; Gan J. Y.; Chin T. S.; Shun T. T.; Tsau C. H.; Chang S. Y. Nanostructured high-entropy alloys with multiple principal elements: Novel alloy design concepts and outcomes. Adv. Eng. Mater. 2004, 6, 299–303. 10.1002/adem.200300567.

[ref13] Cantor B.; Chang I. T. H.; Knight P.; Vincent A. J. B. Microstructural development in equiatomic multicomponent alloys. Mater. Sci. Eng., A 2004, 375–377, 213–218. 10.1016/j.msea.2003.10.257.

[ref14] Pedersen J. K.; Batchelor T. A. A.; Bagger A.; Rossmeisl J. High-entropy alloys as catalysts for the CO_2_ and CO reduction reactions. ACS Catal. 2020, 10, 2169–2176. 10.1021/acscatal.9b04343.

[ref15] Yang J. X.; Dai B. H.; Chiang C. Y.; Chiu I. C.; Pao C. W.; Lu S. Y.; Tsao I. Y.; Lin S. T.; Chiu C. T.; Yeh J. W.; et al. Rapid fabrication of high-entropy ceramic nanomaterials for catalytic reactions. ACS Nano 2021, 15, 12324–12333. 10.1021/acsnano.1c04259. 34269062

[ref16] Feng G.; Ning F.; Song J.; Shang H.; Zhang K.; Ding Z.; Gao P.; Chu W.; Xia D. Sub-2 nm Ultrasmall high-entropy alloy nanoparticles for extremely superior electrocatalytic hydrogen evolution. J. Am. Chem. Soc. 2021, 143, 17117–17127. 10.1021/jacs.1c07643. 34554733

[ref17] Zhu F.; Xu K.; He F.; Xu Y.; Du Z.; Zhang H.; Zeng D.; Liu Y.; Wang H.; Ding D.; Zhou Y.; Chen Y. An active and contaminants-tolerant high-entropy electrode for ceramic fuel cells. ACS Energy Lett. 2024, 9, 556–567. 10.1021/acsenergylett.4c00037.

[ref18] Park N.-Y.; Ryu H.-H.; Kuo L.-Y.; Kaghazchi P.; Yoon C. S.; Sun Y.-K. High-energy cathodes via precision microstructure tailoring for next-generation electric vehicles. ACS Energy Lett. 2021, 6, 4195–4202. 10.1021/acsenergylett.1c02281.

[ref19] Yan P.; Xiao L.; Zheng J.; Zhou Y.; He Y.; Zu X.; Mao S. X.; Xiao J.; Gao F.; Zhang J.-G.; Wang C.-M. Probing the degradation mechanism of Li_2_MnO_3_ cathode for Li-Ion batteries. Chem. Mater. 2015, 27, 975–982. 10.1021/cm504257m.

[ref20] Song J.; Li B.; Chen Y.; Zuo Y.; Ning F.; Shang H.; Feng G.; Liu N.; Shen C.; Ai X.; Xia D. A High-Performance Li-Mn-O Li-rich Cathode material with rhombohedral symmetry via intralayer Li/Mn disordering. Adv. Mater. 2020, 32, 2000190 10.1002/adma.202000190. 32130749

[ref21] Wei S.; Kim S. J.; Kang J.; Zhang Y.; Zhang Y.; Furuhara T.; Park E. S.; Tasan C. C. Natural-mixing guided design of refractory high-entropy alloys with as-cast tensile ductility. Nat. Mater. 2020, 19, 1175–1181. 10.1038/s41563-020-0750-4. 32839590

[ref22] Jiang B.; Yu Y.; Chen H.; Cui J.; Liu X.; Xie L.; He J. Entropy engineering promotes thermoelectric performance in p-type chalcogenides. Nat. Commun. 2021, 12, 3234 10.1038/s41467-021-23569-z. 34050188 PMC8163856

[ref23] Wang X.; Yao H.; Zhang Z.; Li X.; Chen C.; Yin L.; Hu K.; Yan Y.; Li Z.; Yu B.; Cao F.; Liu X.; Lin X.; Zhang Q. Enhanced thermoelectric performance in high entropy alloys Sn_(0.25)_Pb_(0.25)_Mn_(0.25)_Ge_(0.25)_Te. ACS Appl. Mater. Interfaces 2021, 13, 18638–18647. 10.1021/acsami.1c00221. 33847476

[ref24] Sahlberg M.; Karlsson D.; Zlotea C.; Jansson U. Superior hydrogen storage in high entropy alloys. Sci. Rep. 2016, 6, 36770 10.1038/srep36770. 27829659 PMC5103184

[ref25] Marques F.; Balcerzak M.; Winkelmann F.; Zepon G.; Felderhoff M. Review and outlook on high-entropy alloys for hydrogen storage. Energy Environ. Sci. 2021, 14, 5191–5227. 10.1039/D1EE01543E.

[ref26] Gludovatz B.; Hohenwarter A.; Catoor D.; Chang E. H.; George E. P.; Ritchie R. O. A fracture-resistant high-entropy alloy for cryogenic applications. Science 2014, 345, 1153–1158. 10.1126/science.1254581. 25190791

[ref27] Kar N.; McCoy M.; Wolfe J.; Bueno S. L. A.; Shafei I. H.; Skrabalak S. E. Retrosynthetic design of core-shell nanoparticles for thermal conversion to monodisperse high-entropy alloy nanoparticles. Nat. Synth 2024, 3, 175–184. 10.1038/s44160-023-00409-0.

[ref28] Schweidler S.; Brezesinski T.; Breitung B. Entropy-assisted epitaxial coating. Nat. Energy 2024, 9, 240–241. 10.1038/s41560-024-01468-z.

[ref29] Zhu L.; Wang Y.; Chen J.; Li W.; Wang T.; Wu J.; Han S.; Xia Y.; Wu Y.; Wu M.; Wang F.; Zheng Y.; Peng L.; Liu J.; Chen L.; Tang W. Enhancing ionic conductivity in solid electrolyte by relocating diffusion ions to under-coordination sites. Sci. Adv. 2022, 8, eabj7698 10.1126/sciadv.abj7698. 35302845 PMC8932667

[ref30] Zhang R. Z.; Gucci F.; Zhu H.; Chen K.; Reece M. J. Data-driven design of ecofriendly thermoelectric high-entropy sulfides. Inorg. Chem. 2018, 57, 13027–13033. 10.1021/acs.inorgchem.8b02379. 30256098

[ref31] Troparevsky M. C.; Morris J. R.; Kent P. R. C.; Lupini A. R.; Stocks G. M. Criteria for predicting the formation of single-phase high-entropy alloys. Phys. Rev. X 2015, 5, 011041 10.1103/PhysRevX.5.011041.

[ref32] McCormick C. R.; Schaak R. E. Simultaneous multication exchange pathway to high-entropy metal sulfide nanoparticles. J. Am. Chem. Soc. 2021, 143, 1017–1023. 10.1021/jacs.0c11384. 33405919

[ref33] Nemani S. K.; Zhang B.; Wyatt B. C.; Hood Z. D.; Manna S.; Khaledialidusti R.; Hong W.; Sternberg M. G.; Sankaranarayanan S.; Anasori B. High-entropy 2D carbide MXenes: TiVNbMoC_3_ and TiVCrMoC_3_. ACS Nano 2021, 15, 12815–12825. 10.1021/acsnano.1c02775. 34128649

[ref34] Theibault M. J.; McCormick C. R.; Lang S.; Schaak R. E.; Abruna H. D. High entropy sulfide nanoparticles as lithium polysulfide redox catalysts. ACS Nano 2023, 17, 18402–18410. 10.1021/acsnano.3c05869. 37717254

[ref35] Wang R.; Huang J.; Zhang X.; Han J.; Zhang Z.; Gao T.; Xu L.; Liu S.; Xu P.; Song B. Two-dimensional high-entropy metal phosphorus trichalcogenides for enhanced hydrogen evolution reaction. ACS Nano 2022, 16, 3593–3603. 10.1021/acsnano.2c01064. 35212217

[ref36] Stenzel D.; Issac I.; Wang K.; Azmi R.; Singh R.; Jeong J.; Najib S.; Bhattacharya S. S.; Hahn H.; Brezesinski T.; Schweidler S.; Breitung B. High entropy and low symmetry: Triclinic high-entropy molybdates. Inorg. Chem. 2021, 60, 115–123. 10.1021/acs.inorgchem.0c02501. 33314913

[ref37] Du J.; Liu S.; Liu Y.; Wu G.; Liu X.; Zhang W.; Zhang Y.; Hong X.; Li Q.; Kang L. One-dimensional high-entropy compounds. J. Am. Chem. Soc. 2024, 146, 8464–8471. 10.1021/jacs.3c14510. 38483268

[ref38] Rost C. M.; Sachet E.; Borman T.; Moballegh A.; Dickey E. C.; Hou D.; Jones J. L.; Curtarolo S.; Maria J. P. Entropy-stabilized oxides. Nat. Commun. 2015, 6, 8485 10.1038/ncomms9485. 26415623 PMC4598836

[ref39] Iwase K.; Honma I. High-entropy spinel oxide nanoparticles synthesized via supercritical hydrothermal processing as xygen evolution electrocatalysts. ACS Appl. Energy Mater. 2022, 5, 9292–9296. 10.1021/acsaem.2c01751.

[ref40] Aamlid S. S.; Oudah M.; Rottler J.; Hallas A. M. Understanding the role of entropy in high entropy oxides. J. Am. Chem. Soc. 2023, 145, 5991–6006. 10.1021/jacs.2c11608. 36881986

[ref41] Samira S.; Camayang J. C. A.; Patel K.; Gu X.-K.; Nikolla E. Modulating catalytic properties of targeted metal cationic centers in nonstochiometric mixed metal oxides for electrochemical oxygen reduction. ACS Energy Lett. 2021, 6, 1065–1072. 10.1021/acsenergylett.1c00102.

[ref42] Sharp I. D.; Cooper J. K.; Toma F. M.; Buonsanti R. Bismuth vanadate as a platform for accelerating discovery and development of complex transition-metal oxide photoanodes. ACS Energy Lett. 2017, 2, 139–150. 10.1021/acsenergylett.6b00586.

[ref43] Wang L.; Yue S.; Zhang Q.; Zhang Y.; Li Y. R.; Lewis C. S.; Takeuchi K. J.; Marschilok A. C.; Takeuchi E. S.; Wong S. S. Morphological and chemical tuning of high-energy-density metal oxides for lithium ion battery electrode applications. ACS Energy Lett. 2017, 2, 1465–1478. 10.1021/acsenergylett.7b00222.

[ref44] Yeh J.-W.; Chen S.-K.; Lin S.-J.; Gan J.-Y.; Chin T.-S.; Shun T.-T.; Tsau C.-H.; Chang S.-Y. Nanostructured high-entropy alloys with multiple principal elements novel alloy design. Adv. Eng. Mater. 2004, 6, 299–303. 10.1002/adem.200300567.

[ref45] Luan H.-W.; Shao Y.; Li J.-F.; Mao W.-L.; Han Z.-D.; Shao C.; Yao K.-F. Phase stabilities of high entropy alloys. Scr. Mater. 2020, 179, 40–44. 10.1016/j.scriptamat.2019.12.041.

[ref46] Chatterjee A.; Ganguly D.; Sundara R.; Bhattacharya S. S. High-entropy cubic perovskite oxide-based solid electrolyte in quasi-solid-state Li-S Battery. Energy Technol. 2024, 12, 2300576 10.1002/ente.202300576.

[ref47] Dong Q.; Hong M.; Gao J.; Li T.; Cui M.; Li S.; Qiao H.; Brozena A. H.; Yao Y.; Wang X.; Chen G.; Luo J.; Hu L. Rapid synthesis of high-entropy oxide microparticles. Small 2022, 18, 2104761 10.1002/smll.202104761. 35049145

[ref48] Yao Y.; Huang Z.; Xie P.; Lacey S. D.; Jacob R. J.; Xie H.; Chen F.; Nie A.; Pu T.; Rehwoldt M.; Yu D.; Zachariah M. R.; Wang C.; Shahbazian-Yassar R.; Li J.; Hu L. Carbothermal shock synthesis ofhigh-entropy-alloy nanoparticles. Science 2018, 359, 1489–1494. 10.1126/science.aan5412. 29599236

[ref49] Sarkar A.; Wang D.; Kante M. V.; Eiselt L.; Trouillet V.; Iankevich G.; Zhao Z.; Bhattacharya S. S.; Hahn H.; Kruk R. High entropy approach to engineer strongly correlated functionalities in manganites. Adv. Mater. 2023, 35, 2207436 10.1002/adma.202207436. 36383029

[ref50] Balcerzak M.; Kawamura K.; Bobrowski R.; Rutkowski P.; Brylewski T. Mechanochemical synthesis of (Co,Cu,Mg,Ni,Zn)O high-entropy oxide and its physicochemical properties. J. Electron. Mater. 2019, 48, 7105–7113. 10.1007/s11664-019-07512-z.

[ref51] Okejiri F.; Zhang Z.; Liu J.; Liu M.; Yang S.; Dai S. Room-temperature synthesis of high-entropy perovskite oxide nanoparticle catalysts through ultrasonication-based method. ChemSusChem 2020, 13, 111–115. 10.1002/cssc.201902705. 31721472

[ref52] Qiao H.; Saray M. T.; Wang X.; Xu S.; Chen G.; Huang Z.; Chen C.; Zhong G.; Dong Q.; Hong M.; Xie H.; Shahbazian-Yassar R.; Hu L. Scalable synthesis of high entropy alloy nanoparticles by microwave heating. ACS Nano 2021, 15, 14928–14937. 10.1021/acsnano.1c05113. 34423972

[ref53] Dey G. R.; Soliman S. S.; McCormick C. R.; Wood C. H.; Katzbaer R. R.; Schaak R. E. Colloidal nanoparticles of high entropy materials: Capabilities, challenges, and opportunities in synthesis and characterization. ACS Nanosci Au 2024, 4, 3–20. 10.1021/acsnanoscienceau.3c00049. 38406312 PMC10885327

[ref54] Sen S.; Bera S.; Pradhan N. Maneuvering tellurium chemistry to design metal-telluride heterostructures for diverse applications. Chem. Mater. 2022, 34, 9329–9343. 10.1021/acs.chemmater.2c02652.

[ref55] Sen S.; Bera S.; Guria A. K.; Pradhan N. Change in rate of catalytic growths of nanocrystals catalyst for formation of asymmetric multicomponent heterostructures and their self-assembly. J. Phys. Chem. C 2021, 125, 1923–1928. 10.1021/acs.jpcc.0c10974.

[ref56] McKeever H.; Patil N. N.; Palabathuni M.; Singh S. Functional alkali metal-based ternary chalcogenides: Design, properties, and opportunities. Chem. Mater. 2023, 35, 9833–9846. 10.1021/acs.chemmater.3c01652. 38107194 PMC10720346

[ref57] Roth A. N.; Porter A. P.; Horger S.; Ochoa-Romero K.; Guirado G.; Rossini A. J.; Vela J. Lead-free semiconductors: Phase-evolution and superior stability of multinary tin chalcohalides. Chem. Mater. 2024, 36, 4542–4552. 10.1021/acs.chemmater.4c00209. 38764751 PMC11099925

[ref58] Buonsanti R.; Loiudice A.; Mantella V. Colloidal nanocrystals as precursors and intermediates in solid state reactions for multinary oxide nanomaterials. Acc. Chem. Res. 2021, 54, 754–764. 10.1021/acs.accounts.0c00698. 33492926

[ref59] Zeng Y.; Ouyang B.; Liu J.; Byeon Y.-W.; Cai Z.; Miara L. J.; Wang Y.; Ceder G. High-entropy mechanism to boost ionic conductivity. Science 2022, 378, 1320–1324. 10.1126/science.abq1346. 36548421

[ref60] Loiudice A.; Buonsanti R. Reaction intermediates in the synthesis of colloidal nanocrystals. Nat. Synth 2022, 1, 344–351. 10.1038/s44160-022-00056-x.

[ref61] Bera S.; Shyamal S.; Sen S.; Pradhan N. Insights of crystal growth, nucleation density, and shape modulations in the formation of I-V-VI ternary semiconductor nanoplatelet photoelectrocatalysts. J. Phys. Chem. C 2020, 124, 15607–15615. 10.1021/acs.jpcc.0c03947.

[ref62] Kapuria N.; Patil N. N.; Sankaran A.; Laffir F.; Geaney H.; Magner E.; Scanlon M.; Ryan K. M.; Singh S. Engineering polymorphs in colloidal metal dichalcogenides: precursor-mediated phase control, molecular insights into crystallisation kinetics and promising electrochemical activity. J. Mater. Chem. A 2023, 11, 11341–11353. 10.1039/D2TA09892J.

[ref63] Andaraarachchi H. P.; Thompson M. J.; White M. A.; Fan H.-J.; Vela J. Phase-programmed nanofabrication: Effect of organophosphite precursor reactivity on the evolution of nickel and nickel phosphide nanocrystals. Chem. Mater. 2015, 27, 8021–8031. 10.1021/acs.chemmater.5b03506.

[ref64] Kante M. V.; Weber M. L.; Ni S.; van den Bosch I. C. G.; van der Minne E.; Heymann L.; Falling L. J.; Gauquelin N.; Tsvetanova M.; Cunha D. M.; et al. A high-entropy oxide as high-activity Electrocatalyst for Water Oxidation. ACS Nano 2023, 17, 5329–5339. 10.1021/acsnano.2c08096. 36913300 PMC10061923

[ref65] Zhang D.; Xu S.; Li T.; Zhang M.; Qi J.; Wei F.; Meng Q.; Ren Y.; Cao P.; Sui Y. High-entropy oxides prepared by dealloying method for supercapacitors. ACS Appl. Eng. Mater. 2023, 1, 780–789. 10.1021/acsaenm.2c00198.

[ref66] Ye S.; Zhu J.; Zhu S.; Zhao Y.; Li M.; Huang Z.; Wang H.; He J. Design strategies for perovskite-type high-entropy oxides with applications in optics. ACS Appl. Mater. Interfaces 2023, 15, 47475–47486. 10.1021/acsami.3c09447. 37768322

[ref67] Wang H.; Gao X.; Zhang S.; Mei Y.; Ni L.; Gao J.; Liu H.; Hong N.; Zhang B.; Zhu F.; Deng W.; Zou G.; Hou H.; Cao X.-Y.; Chen H.; Ji X. High-entropy Na-deficient layered oxides for sodium-ion batteries. ACS Nano 2023, 17, 12530–12543. 10.1021/acsnano.3c02290. 37382902

[ref68] Katzbaer R. R.; Dos Santos Vieira F. M.; Dabo I.; Mao Z.; Schaak R. E. Band gap narrowing in a high-entropy spinel oxide semiconductor for enhanced oxygen evolution catalysis. J. Am. Chem. Soc. 2023, 145, 6753–6761. 10.1021/jacs.2c12887. 36920866

[ref69] Li Y.; Bai X.; Yuan D.; Yu C.; San X.; Guo Y.; Zhang L.; Ye J. Cu-based high-entropy two-dimensional oxide as stable and active photothermal catalyst. Nat. Commun. 2023, 14, 3171 10.1038/s41467-023-38889-5. 37264007 PMC10235064

[ref70] Li M.; Mei S.; Zheng Y.; Wang L.; Ye L. High-entropy oxides as photocatalysts for organic conversion. Chem. Commun. 2023, 59, 13478–13481. 10.1039/D3CC04435A. 37880980

[ref71] Zhao J.; Bao J.; Yang S.; Niu Q.; Xie R.; Zhang Q.; Chen M.; Zhang P.; Dai S. Exsolution-dissolution of supported metals on high-entropy Co_3_MnNiCuZnO_x_: Toward sintering-resistant catalysis. ACS Catal. 2021, 11, 12247–12257. 10.1021/acscatal.1c03228.

[ref72] Gazda M.; Miruszewski T.; Jaworski D.; Mielewczyk-Gryń A.; Skubida W.; Wachowski S.; Winiarz P.; Dzierzgowski K.; Łapiński M.; Szpunar I.; Dzik E. Novel class of proton conducting materials—high entropy oxides. ACS Materials Lett. 2020, 2, 1315–1321. 10.1021/acsmaterialslett.0c00257.

[ref73] Jin Z.; Lyu J.; Zhao Y.-L.; Li H.; Lin X.; Xie G.; Liu X.; Kai J.-J.; Qiu H.-J. Rugged high-entropy alloy nanowires with in situ formed surface spinel oxide as highly stable electrocatalyst in Zn-air batteries. ACS Materials Lett. 2020, 2, 1698–1706. 10.1021/acsmaterialslett.0c00434.

[ref74] Fracchia M.; Ghigna P.; Pozzi T.; Anselmi Tamburini U.; Colombo V.; Braglia L.; Torelli P. Stabilization by configurational entropy of the Cu(II) active site during CO oxidation on Mg_(0.2)_Co_(0.2)_Ni_(0.2)_Cu_(0.2)_Zn_(0.2)_O. J. Phys. Chem. Lett. 2020, 11, 3589–3593. 10.1021/acs.jpclett.0c00602. 32309955 PMC8007101

[ref75] Zhang Y.; Dai W.; Zhang P.; Lu T.; Pan Y. In-situ electrochemical tuning of (CoNiMnZnFe)_3_O_3.2_ high-entropy oxide for efficient oxygen evolution reactions. J. Alloys Compd. 2021, 868, 159064 10.1016/j.jallcom.2021.159064.

[ref76] Ward-O’Brien B.; McNaughter P. D.; Cai R.; Chattopadhyay A.; Flitcroft J. M.; Smith C. T.; Binks D. J.; Skelton J. M.; Haigh S. J.; Lewis D. J. Quantum confined high-entropy lanthanide oxysulfide colloidal nanocrystals. Nano Lett. 2022, 22, 8045–8051. 10.1021/acs.nanolett.2c01596. 36194549 PMC9614967

[ref77] Guo R.; He T. High-entropy perovskite electrolyte for protonic ceramic fuel cells operating below 600 °C. ACS Materials Lett. 2022, 4, 1646–1652. 10.1021/acsmaterialslett.2c00542.

[ref78] Akrami S.; Murakami Y.; Watanabe M.; Ishihara T.; Arita M.; Fuji M.; Edalati K. Defective high-entropy oxide photocatalyst with high activity for CO_2_ conversion. Appl. Catal. B: Environ. 2022, 303, 120896 10.1016/j.apcatb.2021.120896.

[ref79] Talluri B.; Yoo K.; Kim J. High entropy spinel metal oxide (CoCrFeMnNi)_3_O_4_ nanoparticles as novel efficient electrocatalyst for methanol oxidation and oxygen evolution reactions. J. Environ. Chem. Eng. 2022, 10, 106932 10.1016/j.jece.2021.106932.

[ref80] Wu H.; Lu Q.; Li Y.; Wang J.; Li Y.; Jiang R.; Zhang J.; Zheng X.; Han X.; Zhao N.; et al. Rapid joule-heating synthesis for manufacturing high-entropy oxides as efficient electrocatalysts. Nano Lett. 2022, 22, 6492–6500. 10.1021/acs.nanolett.2c01147. 35950973

[ref81] Gu Y.; Bao A.; Wang X.; Chen Y.; Dong L.; Liu X.; Pan H.; Li Y.; Qi X. Engineering the oxygen vacancies of rocksalt-type high-entropy oxides for enhanced electrocatalysis. Nanoscale 2022, 14, 515–524. 10.1039/D1NR07000B. 34918723

[ref82] Coduri M.; Magnaghi L. R.; Fracchia M.; Biesuz R.; Anselmi-Tamburini U. Assessing phase stability in high-entropy materials by design of experiments: The Case of the (Mg,Ni,Co,Cu,Zn)O System. Chem. Mater. 2024, 36, 720–729. 10.1021/acs.chemmater.3c02120.

[ref83] Leng H.; Zhang P.; Wu J.; Xu T.; Deng H.; Yang P.; Wang S.; Qiu J.; Wu Z.; Li S. The elemental pegging effect in locally ordered nanocrystallites of high-entropy oxide enables superior lithium storage. Nanoscale 2023, 15, 19139–19147. 10.1039/D3NR04006B. 37933578

[ref84] Shi Y.; Ni N.; Ding Q.; Zhao X. Tailoring high-temperature stability and electrical conductivity of high entropy lanthanum Manganite for solid oxide fuel cell cathodes. J. Mater. Chem. A 2022, 10, 2256–2270. 10.1039/D1TA07275G.

[ref85] Ward-O’Brien B.; Pickering E. J.; Ahumada-Lazo R.; Smith C.; Zhong X. L.; Aboura Y.; Alam F.; Binks D. J.; Burnett T. L.; Lewis D. J. Synthesis of high entropy lanthanide oxysulfides via the thermolysis of a molecular precursor cocktail. J. Am. Chem. Soc. 2021, 143, 21560–21566. 10.1021/jacs.1c08995. 34923815

[ref86] Dong Y.; Ren K.; Wang Q.; Shao G.; Wang Y. Interaction of multicomponent disilicate (Yb_0.2_Y_0.2_Lu_0.2_Sc_0.2_Gd_0.2_)_2_Si_2_O_7_ with molten calcia-magnesia-aluminosilicate. J. Adv. Ceram. 2022, 11, 66–74. 10.1007/s40145-021-0517-7.

[ref87] Zhao Z.; Chen H.; Xiang H.; Dai F.-Z.; Wang X.; Xu W.; Sun K.; Peng Z.; Zhou Y. High-entropy (Y_0.2_Nd_0.2_Sm_0.2_Eu_0.2_Er_0.2_)AlO_3_: A promising thermal/environmental barrier material for oxide/oxide composites. J. Mater. Sci. Technol. 2020, 47, 45–51. 10.1016/j.jmst.2020.02.011.

[ref88] Tu T.-Z.; Liu J.-X.; Zhou L.; Liang Y.; Zhang G.-J. Graceful behavior during CMAS corrosion of a high-entropy rare-earth zirconate for thermal barrier coating material. J. Eur. Ceram. Soc. 2022, 42, 649–657. 10.1016/j.jeurceramsoc.2021.10.022.

[ref89] Zhang D.; Yu Y.; Feng X.; Tian Z.; Song R. Thermal barrier coatings with high-entropy oxide as a top coat. Ceram. Int. 2022, 48, 1349–1359. 10.1016/j.ceramint.2021.09.219.

[ref90] Pu Y.; Zhang Q.; Li R.; Chen M.; Du X.; Zhou S. Dielectric properties and electrocaloric effect of high-entropy (Na_0.2_Bi_0.2_Ba_0.2_Sr_0.2_Ca_0.2_)TiO_3_ ceramic. Appl. Phys. Lett. 2019, 115, 223901 10.1063/1.5126652.

[ref91] Wang S.-Y.; Chen T.-Y.; Kuo C.-H.; Lin C.-C.; Huang S.-C.; Lin M.-H.; Wang C.-C.; Chen H.-Y. Operando synchrotron transmission X-ray microscopy study on (Mg, Co, Ni, Cu, Zn)O high-entropy oxide anodes for lithium-ion batteries. Mater. Chem. Phys. 2021, 274, 125105 10.1016/j.matchemphys.2021.125105.

[ref92] Yang W.; Zheng G. High energy storage density and efficiency in nanostructured (Bi_0.2_Na_0.2_K_0.2_La_0.2_Sr_0.2_)TiO_3_ high-entropy ceramics. J. Am. Ceram. Soc. 2022, 105, 1083–1094. 10.1111/jace.18129.

[ref93] Chen Y.-T.; Lee J.-T.; Liang T. Y.; Song Y.-H.; Wu J. M. Solid composite electrolyte based on oxygen vacancy effect of Li_x_(CoCrFeMnNi)O_4-y_ high entropy oxides. Electrochim. Acta 2023, 456, 142459 10.1016/j.electacta.2023.142459.

[ref94] Bai Y.; Li J.; Lu H.; Liu J.; Ma C.; Wang B.; Zhao X.; Deng J. Ultrafast high-temperature sintering of high-entropy oxides with refined microstructure and superior lithium-ion storage performance. J. Adv. Ceram. 2023, 12, 1857–1871. 10.26599/JAC.2023.9220793.

[ref95] Sarkar A.; Mannava P. K.; Velasco L.; Das C.; Breitung B.; Bhattacharya S. S.; Kruk R.; Hahn H. Determining role of individual cations in high entropy oxides: Structure and reversible tuning of optical properties. Scr. Mater. 2022, 207, 114273 10.1016/j.scriptamat.2021.114273.

[ref96] Yang H.; Lin G.; Bu H.; Liu H.; Yang L.; Wang W.; Lin X.; Fu C.; Wang Y.; Zeng C. Single-phase forming ability of high-entropy ceramics from a size disorder perspective: A case study of (La_0.2_Eu_0.2_Gd_0.2_Y_0.2_Yb_0.2_)_2_Zr_2_O_7_. Ceram. Int. 2022, 48, 6956–6965. 10.1016/j.ceramint.2021.11.252.

[ref97] Shi Y.; Xu Q.; Tian Z.; Liu G.; Ma C.; Zheng W. Ionic liquid-hydroxide-mediated low-temperature synthesis of high-entropy perovskite oxide nanoparticles. Nanoscale 2022, 14, 7817–7827. 10.1039/D1NR08316C. 35262130

[ref98] Yang K.; Bryce K.; Zhu W.; Zhao D.; Lian J. Multicomponent pyrochlore solid solutions with uranium incorporation - A new perspective of materials design for nuclear applications. J. Eur. Ceram. Soc. 2021, 41, 2870–2882. 10.1016/j.jeurceramsoc.2020.12.012.

[ref99] Zhang M.; Xu X.; Yue Y.; Palma M.; Reece M. J.; Yan H. Multi elements substituted Aurivillius phase relaxor ferroelectrics using high entropy design concept. Mater. Des. 2021, 200, 109447 10.1016/j.matdes.2020.109447.

[ref100] Xiang H.; Yao L.; Chen J.; Yang A.; Yang H.; Fang L. Microwave dielectric high-entropy ceramic Li(Gd_0.2_Ho_0.2_Er_0.2_Yb_0.2_Lu_0.2_)GeO_4_ with stable temperature coefficient for low-temperature cofired ceramic technologies. J. Mater. Sci. Technol. 2021, 93, 28–32. 10.1016/j.jmst.2021.03.057.

[ref101] Jing L.; Li W.; Gao C.; Li M.; Fei W. Enhanced energy storage performance achieved in multilayered PVDF-PMMA nanocomposites incorporated with high-entropy oxide nanofibers. ACS Appl. Energy Mater. 2023, 6, 3093–3101. 10.1021/acsaem.3c00054.

[ref102] Wang B.; Wang J.; Yao J.; Chang A. An innovative approach to design highly stabilized thermistor materials: dual-phase five-component CoMnFeZnYO_7_ ceramics. J. Mater. Chem. C 2021, 9, 1794–1803. 10.1039/D0TC05422D.

[ref103] Zhou Z.; Xue L.; Yang F.; Jiang Z.; Wang K.; Xie M.; Chen H. Promising rare-earth high-entropy ceramic driving foamed silicon rubber composite to reduce 100–300 Hz low-frequency noise. Mater. Lett. 2022, 313, 131824 10.1016/j.matlet.2022.131824.

[ref104] Salian A.; Pujar P.; Vardhan R. V.; Cho H.; Kim S.; Mandal S. Evolution of high dielectric permittivity in low-temperature solution combustion-processed phase-pure high entropy oxide (CoMnNiFeCr)O for thin film transistors. ACS Appl. Electron. Mater. 2023, 5, 2608–2623. 10.1021/acsaelm.3c00094.

[ref105] Wu P. Y.; Le K. T.; Lin H. Y.; Chen Y. C.; Wu P. H.; Wu J. M. Flexoelectric catalysts based on hierarchical wrinkling surface of centrosymmetric high-entropy oxide. ACS Nano 2023, 17, 17417–17426. 10.1021/acsnano.3c05478. 37581913

[ref106] Qiu N.; Chen H.; Yang Z.; Sun S.; Wang Y.; Cui Y. A high entropy oxide (Mg0.2Co0.2Ni0.2Cu0.2Zn0.2O) with superior lithium storage performance. J. Alloys Compd. 2019, 777, 767–774. 10.1016/j.jallcom.2018.11.049.

[ref107] Wang Y.-Q.; Wang H.-M.; Jiang Y.-C.; Li G.-R.; Liu S.; Gao X.-P. High-entropy oxide nanofibers as catalytic host promising high volumetric capacity of sulfur-based composites for lithium-sulfur batteries. ACS Appl. Energy Mater. 2023, 6, 8377–8387. 10.1021/acsaem.3c01087.

[ref108] Shen K.; Wang T.; Li C.; Chen M.; Niu L.; Gong Y. Designing highly-efficient oxygen evolution reaction FeCoNiCrMnO_x_ electrocatalyst via coexisted crystalline and amorphous phases: experiment and theory. Appl. Surf. Sci. 2024, 650, 159102 10.1016/j.apsusc.2023.159102.

[ref109] Riley C.; De La Riva A.; Park J. E.; Percival S. J.; Benavidez A.; Coker E. N.; Aidun R. E.; Paisley E. A.; Datye A.; Chou S. S. A high entropy oxide designed to catalyze CO oxidation without precious metals. ACS Appl. Mater. Interfaces 2021, 13, 8120–8128. 10.1021/acsami.0c17446. 33565850

[ref110] Su L.; Chen X.; Xu L.; Eldred T.; Smith J.; DellaRova C.; Wang H.; Gao W. Visualizing the formation of high-entropy fluorite oxides from an amorphous precursor at atomic resolution. ACS Nano 2022, 16, 21397–21406. 10.1021/acsnano.2c09760. 36454037

[ref111] Hanabata S.; Kusada K.; Yamamoto T.; Toriyama T.; Matsumura S.; Kawaguchi S.; Kubota Y.; Nishida Y.; Haneda M.; Kitagawa H. Denary high-entropy oxide nanoparticles synthesized by a continuous supercritical hydrothermal flow process. J. Am. Chem. Soc. 2024, 146, 181–186. 10.1021/jacs.3c07351. 38153046

[ref112] Johnstone G. H. J.; Gonzalez-Rivas M. U.; Taddei K. M.; Sutarto R.; Sawatzky G. A.; Green R. J.; Oudah M.; Hallas A. M. Entropy engineering and tunable magnetic order in the spinel high-entropy oxide. J. Am. Chem. Soc. 2022, 144, 20590–20600. 10.1021/jacs.2c06768. 36321637

[ref113] Kirsch A.; Bojesen E. D.; Lefeld N.; Larsen R.; Mathiesen J. K.; Skjaervo S. L.; Pittkowski R. K.; Sheptyakov D.; Jensen K. M. O. High-entropy oxides in the mullite-type structure. Chem. Mater. 2023, 35, 8664–8674. 10.1021/acs.chemmater.3c01830. 37901145 PMC10601478

[ref114] Katzbaer R. R.; Vincent W. M.; Mao Z.; Schaak R. E. Synthesis and magnetic, optical, and electrocatalytic properties of high-entropy mixed-metal tungsten and molybdenum oxides. Inorg. Chem. 2023, 62, 7843–7852. 10.1021/acs.inorgchem.3c00541. 37163751

[ref115] Zhang M.; Ye J.; Gao Y.; Duan X.; Zhao J.; Zhang S.; Lu X.; Luo K.; Wang Q.; Niu Q.; Zhang P.; Dai S. General synthesis of high-entropy oxide nanofibers. ACS Nano 2024, 18, 1449–1463. 10.1021/acsnano.3c07506. 38175529

[ref116] Jiang Y.; Liang Z.; Fu H.; Sun M.; Wang S.; Huang B.; Du Y. Pt-Modified high entropy rare earth oxide for efficient hydrogen evolution in pH-universal environments. J. Am. Chem. Soc. 2024, 146, 9012–9025. 10.1021/jacs.3c13367. 38516778

[ref117] Han Y.; Liu X.; Zhang Q.; Huang M.; Li Y.; Pan W.; Zong P. A.; Li L.; Yang Z.; Feng Y.; et al. Ultra-dense dislocations stabilized in high entropy oxide ceramics. Nat. Commun. 2022, 13, 2871 10.1038/s41467-022-30260-4. 35610224 PMC9130511

[ref118] Krysko E.; Min L.; Wang Y.; Zhang N.; Barber J. P.; Niculescu G. E.; Wright J. T.; Li F.; Burrage K.; Matsuda M.; Robinson R. A.; Zhang Q.; Katzbaer R.; Schaak R.; Terrones M.; Rost C. M.; Mao Z. Studies on the structure and the magnetic properties of high-entropy spinel oxide (MgMnFeCoNi)Al_2_O_4_. APL Mater. 2023, 11, 101123 10.1063/5.0161401.

[ref119] Nguyen T. X.; Su Y. H.; Hattrick-Simpers J.; Joress H.; Nagata T.; Chang K. S.; Sarker S.; Mehta A.; Ting J. M. Exploring the first high-entropy thin film libraries: Composition spread-controlled crystalline structure. ACS Comb. Sci. 2020, 22, 858–866. 10.1021/acscombsci.0c00159. 33146510 PMC8415495

[ref120] Spurling R. J.; Lass E. A.; Wang X.; Page K. Entropy-driven phase transitions in complex ceramic oxides. Phys. Rev. Materials. 2022, 6, 090301 10.1103/PhysRevMaterials.6.090301.

[ref121] Sun Y.; Dai S. High-entropy materials for catalysis: A new frontier. Science Advances 2021, 7, eabg1600 10.1126/sciadv.abg1600. 33980494 PMC8115918

[ref122] He H.; Kou P.; Zhang Z.; Wang D.; Zheng R.; Sun H.; Liu Y.; Wang Z. Coupling high entropy oxide with hollow carbon spheres by rapid microwave solvothermal strategy for boosting oxygen evolution reaction. J. Colloid Interface Sci. 2024, 653, 179–188. 10.1016/j.jcis.2023.09.063. 37713916

[ref123] Mehrabi-Kalajahi S.; Ostovari Moghaddam A.; Hadavimoghaddam F.; Salari R.; Varfolomeev M. A.; Zinnatullin A. L.; Minnebaev K. R.; Emelianov D. A.; Uchaev D. A.; Fereidonnejad R.; Zaitseva O.; Khasanova G. R.; Trofimov E. A.; Rozhenko A.; Cabot A.; Vagizov F. G. (CoFeMnCuNiCr)_3_O_4_ High-entropy oxide nanoparticles immobilized on reduced graphene oxide as heterogeneous catalysts for solvent-free aerobic oxidation of benzyl alcohol. ACS Appl. Nano Mater. 2024, 7, 5513–5524. 10.1021/acsanm.4c00103.

[ref124] Sarkar A.; Velasco L.; Wang D.; Wang Q.; Talasila G.; de Biasi L.; Kubel C.; Brezesinski T.; Bhattacharya S. S.; Hahn H.; et al. High entropy oxides for reversible energy storage. Nat. Commun. 2018, 9, 3400 10.1038/s41467-018-05774-5. 30143625 PMC6109100

[ref125] Yang X.; Wang H.; Song Y.; Liu K.; Huang T.; Wang X.; Zhang C.; Li J. Low-temperature synthesis of a porous high-entropy transition-metal oxide as an anode for high-performance lithium-Ion Batteries. ACS Appl. Mater. Interfaces 2022, 14, 26873–26881. 10.1021/acsami.2c07576. 35653293

[ref126] Hou S.; Su L.; Wang S.; Cui Y.; Cao J.; Min H.; Bao J.; Shen Y.; Zhang Q.; Sun Z.; Zhu C.; Chen J.; Zhang Q.; Xu F. Unlocking the origins of highly reversible lithium storage and stable cycling in a spinel high-entropy oxide anode for lithium-ion batteries. Adv. Funct. Mater. 2024, 34, 2307923 10.1002/adfm.202307923.

[ref127] Lun Z.; Ouyang B.; Kwon D. H.; Ha Y.; Foley E. E.; Huang T. Y.; Cai Z.; Kim H.; Balasubramanian M.; Sun Y.; et al. Cation-disordered rocksalt-type high-entropy cathodes for Li-ion batteries. Nat. Mater. 2021, 20, 214–221. 10.1038/s41563-020-00816-0. 33046857

[ref128] Rana J.; Stan M.; Kloepsch R.; Li J.; Schumacher G.; Welter E.; Zizak I.; Banhart J.; Winter M. Structural changes in Li_2_MnO_3_ cathode material for Li-ion batteries. Adv. Energy Mater. 2014, 4, 1300998 10.1002/aenm.201300998.

[ref129] Chen D.; Ahn J.; Chen G. An overview of cation-disordered lithium-excess rocksalt Cathodes. ACS Energy Lett. 2021, 1358–1376. 10.1021/acsenergylett.1c00203.

[ref130] Song J.; Ning F.; Zuo Y.; Li A.; Wang H.; Zhang K.; Yang T.; Yang Y.; Gao C.; Xiao W.; et al. Entropy stabilization strategy for enhancing the local structural adaptability of Li-rich cathode Materials. Adv. Mater. 2023, 35, 2208726 10.1002/adma.202208726. 36385715

[ref131] Han S.; Wang Z.; Ma Y.; Miao Y.; Wang X.; Wang Y.; Wang Y. Fast ion-conducting high-entropy garnet solid-state electrolytes with excellent air stability. J. Adv. Ceram. 2023, 12, 1201–1213. 10.26599/JAC.2023.9220749.

[ref132] Palakkathodi Kammampata S.; Yamada H.; Ito T.; Paul R.; Thangadurai V. The activation entropy for ionic conduction and critical current density for Li charge transfer in novel garnet-type Li_6.5_La_2.9_A_0.1_Zr_1.4_Ta_0.6_O_12_ (A = Ca, Sr, Ba) solid electrolytes. J. Mater. Chem. A 2020, 8, 2581–2590. 10.1039/C9TA12193E.

[ref133] Bonnet E.; Grenier J. C.; Bassat J. M.; Jacob A.; Delatouche B.; Bourdais S. On the ionic conductivity of some zirconia-derived high-entropy oxides. J. Eur. Ceram. Soc. 2021, 41, 4505–4515. 10.1016/j.jeurceramsoc.2021.03.021.

